# Genomic and biological study of fusion genes as resistance mechanisms to EGFR inhibitors

**DOI:** 10.1038/s41467-022-33210-2

**Published:** 2022-09-24

**Authors:** Yoshihisa Kobayashi, Geoffrey R. Oxnard, Elizabeth F. Cohen, Navin R. Mahadevan, Joao V. Alessi, Yin P. Hung, Arrien A. Bertram, David E. Heppner, Mauricio F. Ribeiro, Karina P. Sacardo, Rodrigo Saddi, Mariana P. Macedo, Rafael B. Blasco, Jiaqi Li, Kari J. Kurppa, Tom Nguyen, Emma Voligny, Guruprasad Ananda, Roberto Chiarle, Artur Katz, Michael Y. Tolstorukov, Lynette M. Sholl, Pasi A. Jänne

**Affiliations:** 1 Department of Medical Oncology, Dana-Farber Cancer Institute and Harvard Medical School, Boston, MA 02215 USA; 2 Division of Molecular Pathology, National Cancer Center Research Institute, Tokyo, 1040045 Japan; 3 Lowe Center for Thoracic Oncology, Dana-Farber Cancer Institute, Boston, MA 02215 USA; 4 Department of Informatics and Analytics, Dana-Farber Cancer Institute, Boston, MA 02215 USA; 5 Department of Pathology, Brigham and Women’s Hospital, Boston, MA 02115 USA; 6 Department of Pathology, Massachusetts General Hospital, Boston, MA 02114 USA; 7 Department of Chemistry, University at Buffalo, State University of New York, Buffalo, NY 14260-3000 USA; 8 Department of Pharmacology and Therapeutics, Roswell Park Comprehensive Cancer Center, Buffalo, NY 14263 USA; 9 Department of Medical Oncology, Hospital Sírio-Libanês, São Paulo-SP, 01308-050 Brazil; 10 Department of Pathology, Hospital Sírio-Libanês, São Paulo-SP, 01308-050 Brazil; 11 Department of Pathology, Boston Children’s Hospital, Boston, MA 02115 USA; 12 Institute of Biomedicine, and MediCity Research Laboratories, University of Turku, Turku, 20520 Finland; 13 Department of Data Science, Dana-Farber Cancer Institute, Boston, MA 02215 USA; 14 Department of Molecular Biotechnology and Health Sciences, University of Torino, Torino, 10126 Italy; 15 Belfer Center for Applied Cancer Science, Dana-Farber Cancer Institute, Boston, MA 02215 USA

**Keywords:** Cancer therapeutic resistance, Targeted therapies, Cancer genomics, Non-small-cell lung cancer

## Abstract

The clinical significance of gene fusions detected by DNA-based next generation sequencing remains unclear as resistance mechanisms to EGFR tyrosine kinase inhibitors in *EGFR* mutant non-small cell lung cancer. By studying EGFR inhibitor-resistant patients treated with a combination of an EGFR inhibitor and a drug targeting the putative resistance-causing fusion oncogene, we identify patients who benefit and those who do not from this treatment approach. Through evaluation including RNA-seq of potential drug resistance-imparting fusion oncogenes in 504 patients with *EGFR* mutant lung cancer, we identify only a minority of them as functional, potentially capable of imparting EGFR inhibitor resistance. We further functionally validate fusion oncogenes in vitro using CRISPR-based editing of *EGFR* mutant cell lines and use these models to identify known and unknown drug resistance mechanisms to combination therapies. Collectively, our results partially reveal the complex nature of fusion oncogenes as potential drug resistance mechanisms and highlight approaches that can be undertaken to determine their functional significance.

## Abstract (web summary)

Fusion genes have been proposed as a potential mechanism of resistance to EGFR tyrosine kinase inhibitors (TKIs) in lung cancer. Here, the authors identify gene fusions that are associated with resistance to EGFR TKIs in non-small cell lung cancers, and test how these fusions impact the response to EGFR TKIs in vitro.

## Introduction

Activating mutations in epidermal growth factor receptor (*EGFR*) are detected in up to 20% of lung adenocarcinomas^1^, and the standard treatment for these cancers is the use of EGFR tyrosine kinase inhibitors (TKIs). Despite a robust initial response to TKIs, lung cancers inevitably acquire resistance to these drugs. The major mechanisms of such acquired resistance are secondary *EGFR* mutations^2,3^, bypass signaling through amplification of *MET*^4^, and histological transformation to small cell lung cancer^5^. Osimertinib is a mutant-selective EGFR-TKI that targets treatment-naïve *EGFR* mutant lung cancers and cancers that acquire a gatekeeper *EGFR* T790M mutation after treatment with EGFR-TKIs^6,7^.

Although we and others described oncogenic fusion genes as a mechanism of resistance to osimertinib in 10% of drug resistant cancers, in vitro and clinical data have been limited to the study of only representative genes^8–13^. Targeted DNA-based next generation sequencing (NGS) assays have been approved as companion diagnostics, for use with targeted therapy in lung cancer. Widespread clinical use of this type of NGS could potentially increase the detection of unexpected fusion genes, but the technique has limitations. For example, the majority of fusion breakpoints are located in intronic regions, and thus true fusion partners can be difficult to distinguish from highly homologous regions based on the short fragments of reads. RNA-based targeted NGS can detect unknown fusions only in cases where the specific exons of at least the 5′ or 3′ genes have been pre-designed^14^. However, we have recently shown that bulk RNA sequencing (RNA-seq) offers an unbiased genome-wide method to identify oncogenic fusions that are missed by hybrid capture NGS^15^.

Here we undertake a comprehensive systematic validation of all fusion genes detected by the DNA-based hybrid capture NGS OncoPanel in *EGFR*-mutant lung cancer, as underappreciated mediators of TKI resistance. Suspected fusions are aligned with the clinical response to EGFR-TKIs, validated by RNA-seq, and through CRISPR-Cas9 genome-edited in vitro cell models.

## Results

### Clinical response to combination therapy to overcome fusion-mediated resistance

Prior studies have demonstrated the efficacy of adding a second agent against a putative resistance mechanism in patients with *EGFR* mutant non-small cell lung cancer (NSCLC)^10,16^. Here we present four patients with *EGFR* mutant lung cancer whose drug resistant cancers contained putative fusion oncogenes. In each of the examples, combination therapy was either administered or being considered.

**Fig. 1:**
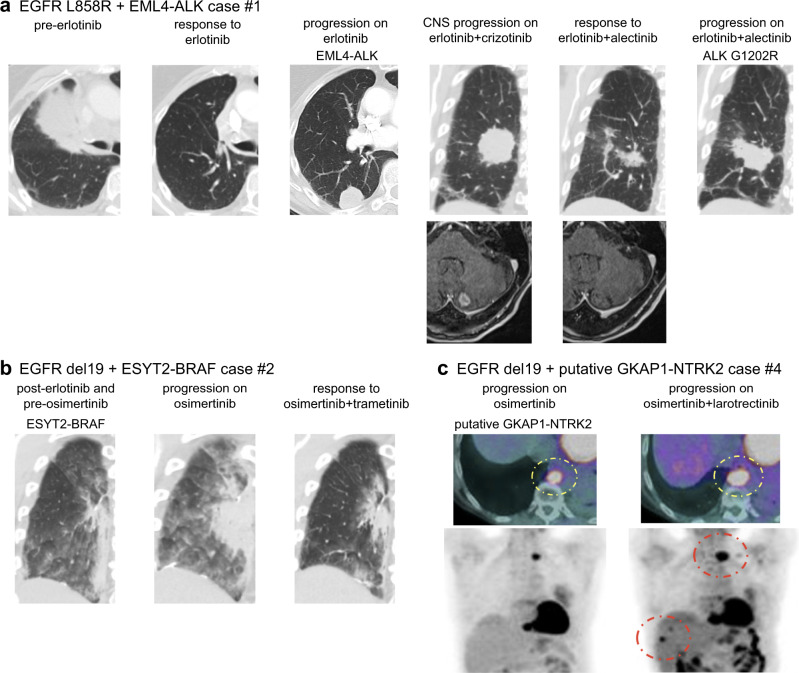
Clinical response to combination therapy aimed at overcoming fusion-mediated drug resistance. **a** *EGFR* L858R mutant adenocarcinoma acquired *ALK* fusion. Combination of erlotinib and alectinib evoked a response in lung and brain lesions. **b** Combined use of osimertinib and trametinib successfully shrank the *EGFR* exon 19 deletion (del19) lung adenocarcinoma, which had acquired an *ESYT2-BRAF* fusion. **c** Positron emission tomography (PET)-CT images show the progression of *EGFR* del19 adenocarcinoma with a putative *GKAP1-NTRK2* fusion following treatment with osimertinib plus larotrectinib.

Case #1 had an *EGFR* L858R mutant adenocarcinoma that was initially negative for ALK expression by immunohistochemistry (IHC). After the successful treatment with erlotinib, *EML4-ALK* fusion (chr2:42477047_2:29446862) was detected by OncoPanel (a targeted NGS panel^17^), and *ALK* rearrangement was confirmed by fluorescence in situ hybridization (FISH). Confirmatory IHC showed expression of both ALK and mutation-specific EGFR-L858R protein in the same tumor cells, despite the generally mutually exclusive occurrences of driver mutations in treatment-naïve lung cancers^18–20^ (Fig. 1a and Supplementary Fig. 1). Co-existence of *EGFR* mutation and *EML4-ALK* was further supported by the following data: first, the variant allele frequency of *EGFR* L858R by NGS were 87% of 965 reads in pretreatment samples and 79% of 927 reads in resistant sample. Second, EGFR *L858R* in pretreatment samples was detected by an allele specific qPCR assay as well. The patient was treated with a combination of crizotinib and erlotinib but developed lung progression and brain metastases 12 months later. Treatment was subsequently switched to a combination of alectinib and erlotinib, which led to a reduction in the lung and the brain lesions. However, progression developed in the lung lesion in 10 months, and NGS of the growing lesion identified *ALK* G1202R, a known alectinib resistance mutation^21^. *ALK* G1202R is known to be sensitive to lorlatinib but this was not clinically available at the time of this patient’s treatment.

Case #2 had an *EGFR* deletion in exon 19 (del19) adenocarcinoma that acquired an *ESYT2-BRAF* fusion (chr7:140481539_7:158559016) and *EGFR* T790M after progression on erlotinib^8^. Although osimertinib did not shrink the tumor, we observed a loss of T790M but *ESYT2-BRAF* was retained as assayed by NGS from a growing tumor. The patient was treated with osimertinib and trametinib, which led to tumor shrinkage (Fig. 1b).

Case #3 was a 29-year-old female with *EGFR* del19 adenocarcinoma. After initial treatment with EGFR-TKI icotinib, *EGFR* T790M mutation developed, and this therapy was followed by osimertinib with tumor shrinkage. OncoPanel detected a loss of T790M, plus an *FGFR1*-intergenic fusion (intron 3 of *FGFR1* and a highly repetitive non-coding region, chr8:38307562_chr8:32056532) within a growing pelvic mass. Further review of the raw NGS data revealed that this fusion was supported by 13 split reads and 2 discordant reads. Three kinds of structural variants callers, BreaKmer, Manta, and SvABA tool, consistently detected this fusion which exclude the possibility of being an artifact and support the real DNA structural variant. To confirm the putative *FGFR1* fusion, we used an RNA-based anchored multiplex PCR for targeted NGS^14^, but did not detect the fusion. Although the plan was to administer osimertinib and an FGFR inhibitor, the treating provider elected not to pursue the combination treatment given the uncertain role of the *FGFR1* fusion as a mechanism of resistance in this patient.

Case #4 is a patient with an *EGFR* del19 adenocarcinoma who was treated with erlotinib and developed *EGFR* T790M. After 4 years of treatment with osimertinib, *GKAP1-NTRK2* fusion (chr9:86395295_chr9:87425455) was detected by hybrid capture RNA-based targeted sequencing (Illumina TruSight™Oncology 500). Based on the fusion call by RNA-based NGS, and on previous reports of *GKAP1-NTRK2* fusion present in gliomas^22–24^, this patient was treated with a combination of osimertinib and larotrectinib. However, tumors did not respond to this combination therapy (Fig. 1c). Further review of the initial RNA-based NGS data revealed that the called *NTRK2* isoform did not have a kinase domain and was shorter than the full-length isoform with a kinase domain (Supplementary Fig. 2). Additionally, transcript reads supporting this fusion presented at low levels of 1.7% (13/765 reads). Taken together, we conclude that the putative *GKAP1-NTRK2* fusion in this case was not a functional oncogenic fusion and did not mediate resistance.

### Comprehensive analyses of all fusions in *EGFR* mutant lung cancer

**Fig. 2:**
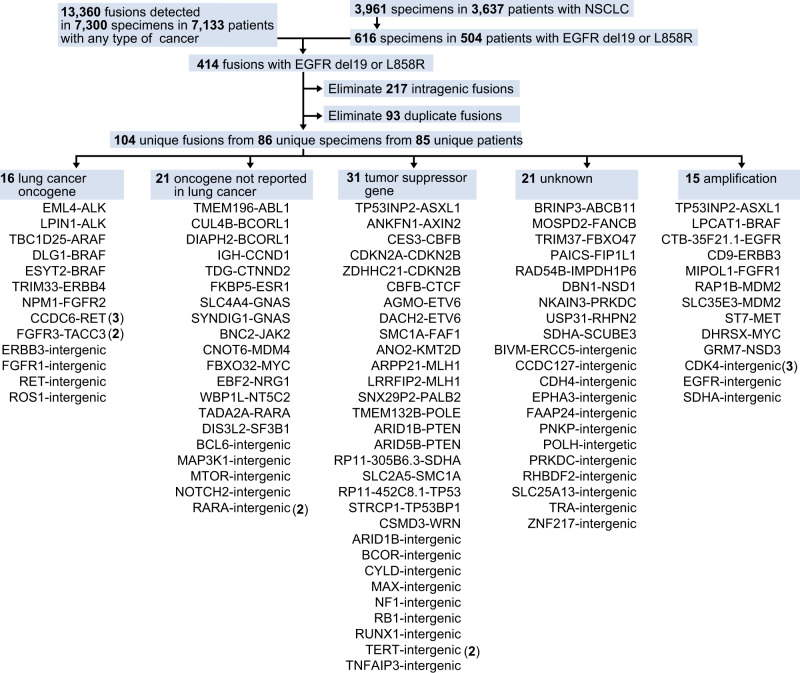
Comprehensive analyses of all fusions in *EGFR* mutant lung cancer. Prospectively collected genetic data on fusions from all cancer patients, and mutational data from patients with non-small cell lung cancer (NSCLC; detected by OncoPanel at Dana Farber Cancer Institute) were combined. Unique fusions identified in patients with *EGFR* L858R or deletion in exon 19 (del19) were classified into five groups.

Based on these clinical examples, we next undertook a study of all putative fusion oncogenes in *EGFR* mutant lung cancer via a comprehensive analysis of 3637 patients with lung cancer. A total of 104 unique fusions in 504 patients with *EGFR* del 19 or L858R mutations were formally reported to clinicians by molecular pathologists based on the level of evidence assessed by the read number, alignment quality, and strandedness of OncoPanel results (Supplementary Table 1). Of these fusions, 16 (including *ALK*, *ARAF*, *BRAF*, *FGFR1*, *FGFR3*, *RET*, and *ROS1*) and 21 (including *ABL1*, *GNAS*, *JAK2*, and *NRG1*) were classified as either related to lung cancer, or as oncogenes that have not been reported in lung cancer, respectively (Fig. 2). We focused on these 37 putative fusions involving established oncogenes.

### Clinical annotation of putative fusions in *EGFR* mutant lung cancer

**Fig. 3:**
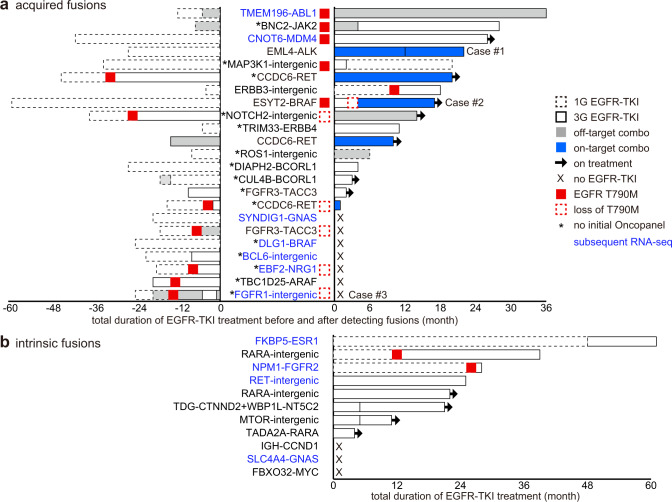
Clinical annotation of putative fusions in *EGFR* mutant lung cancer. **a** Clinical course of patients with fusions, which were detected by DNA-based next generation sequencing (NGS) OncoPanel, following treatment with first or third generation (1G or 3G) EGFR tyrosine kinase inhibitors (TKIs). Asterisks indicate samples that did not undergo initial evaluation by OncoPanel. Bar charts show total durations of EGFR-TKI treatment before and after the fusions were detected. RNA from these samples were submitted for further RNA sequencing (blue). **b** Clinical course of patients with fusions that were detected prior to treatment with EGFR-TKI.

Among 504 patients in this cohort, 263 developed resistance to EGFR inhibitors and 197 of them received biopsy and subsequent evaluation by DNA-based NGS OncoPanel. Putative oncogene-related fusions were detected in 12 % (23/197) of these cases. Total durations of EGFR-TKI treatments before and after detection of fusion genes are summarized (Fig. 3a, b). Patients with *EML4-ALK* (case #1 in Fig. 1a) or *ESYT2-BRAF* (Case #2 in Fig. 1b), as well as 2 patients with *CCDC6-RET* were successfully treated with on-target combination therapy (i.e. combination of an EGFR inhibitor and a drug targeting the putative resistance-causing fusion oncogene). Despite the presence of fusions involving established oncogenes including *ABL1*, *JAK2*, or *FGFR2* with different partner genes, and *RET*-intergenic fusion, these patients maintained disease control with EGFR-TKIs for more than 2 years, suggesting that fusions with these known oncogenes did not lead to clinical drug resistance. To understand this discrepancy and to establish the clinical significance of putative fusions in patients who have not undergone EGFR-TKI treatment, including case #3 with putative *FGFR1* fusion, we performed bulk RNA-sequencing, followed by bioinformatic analyses – this approach previously led us to discover an unknown oncogenic fusion^15^.

### Comparison of putative fusions by RNA-seq and DNA-seq

**Fig. 4:**
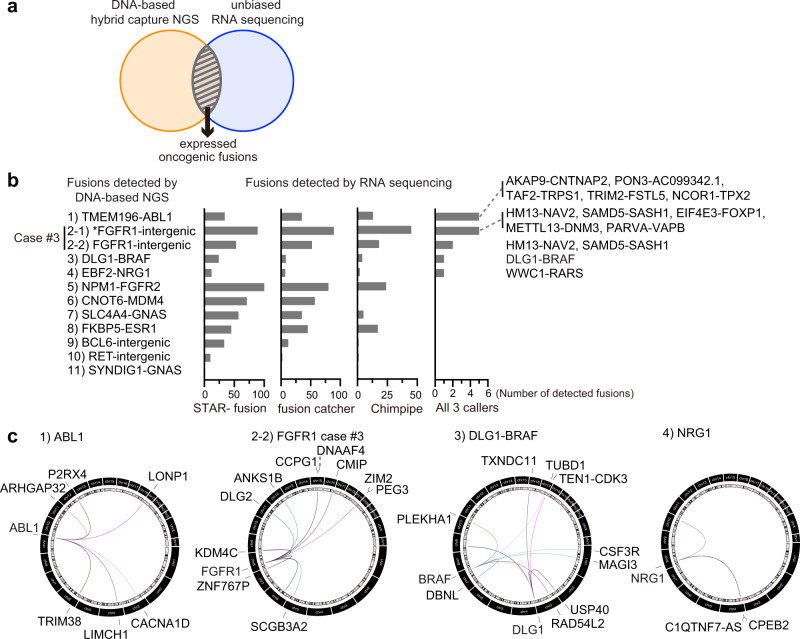
Comparison of putative fusions by DNA-seq and RNA-seq. **a** Schema of integrating data obtained by DNA-based next generation sequencing (NGS) OncoPanel, and RNA-sequencing (RNA-seq), to pick out expressed oncogenic fusions. **b** The candidate fusions detected by each fusion caller from RNA-seq data were aligned with putative oncogenic fusions detected by OncoPanel. Fusions detected by all three fusion callers were noted. Asterisk points to data obtained from the leftover RNA sample from the RNA-based anchored multiplex PCR for targeted NGS. **c** Circos plots showing discordant reads with *ABL1*, *FGFR1*, *BRAF*, *DLG1*, or *NRG1* detected by RNA-seq.

DNA-based NGS assays can detect gene rearrangements of expressed as well as unexpressed fusions. RNA sequencing can help to identify the fusion events that are expressed. Similar to DNA-seq, presence of discordant RNA-seq read-pairs (i.e., pairs where read-mates are not aligned to the reference genome with the expected distance and/or orientation) and reads that are associated with split alignments are indicative of fusions; however only expressed fusions would be detected in RNA-seq data. We thus integrated both types of analyses to select expressed oncogenic fusions (Fig. 4a). We submitted formalin-fixed paraffin-embedded (FFPE) or frozen samples from 11 patients for bulk RNA-sequencing at Broad Institute. In patient #3 with a putative *FGFR1* fusion, both the FFPE sample and the leftover RNA from a previous anchored multiplex PCR for targeted NGS were analyzed to validate samples, given that intron 3 of *FGFR1* (where the breakpoints of putative *FGFR1* fusions in this patient are located) is not pre-designed by this assay. In 12 samples from 11 patients, 3 different fusion callers consistently detected only the *DLG1-BRAF* fusion, which confirmed the OncoPanel data (Fig. 4b; Supplementary Fig. 3a; and Supplementary Table 2). None of other putative fusions detected by OncoPanel including the putative *FGFR1* fusion of patient #3 were validated even when lowering the threshold to detection by 2 fusion callers (Supplementary Table 3). To determine whether potential evidence for the oncogenic fusions could be found in the samples below the thresholds of fusion callers, we examined the raw discordant reads. Although with insufficient level of support to reach statistical significance, the assessment of such reads revealed a wide variety of putative fusion events with oncogenes including *ABL1*. These findings concurred with the fact that the breakpoints of *RET* intron 12, *ABL1* intron 1, or *NRG1* intron 5 that were detected by OncoPanel are commonly reported sites in patients with established fusions such as *CCDC6-RET*, *BCR-ABL1*, or *NRG1* fusion (Fig. 4c and Supplementary Fig. 3b). However, none of previously reported nor OncoPanel-detected fusion partner genes were involved. These observations concur with clinical data that patients were successfully treated with EGFR-TKIs despite the presence of putative *ABL1*, *MDM4*, *ESR1*, *FGFR2*, or *RET* fusions (Fig. 3a, b).

As orthogonal techniques for the validation of putative fusions, RT-PCR and FISH were performed in available clinical samples. RT-PCR and subsequent Sanger sequencing confirmed *DLG1*-*BRAF* fusion which was detected by both DNA-based NGS and RNA-seq. Additionally, very faint *FKBP5-ESR1* band was also detected by RT-PCR which concur with the fact that RNA-seq did not support the enough expression of this fusion due to the fusion breakpoint’s location in the *FKBP5* exon UTR after stop codon, resulting in production of only FKBP5 protein but not fusion protein (Supplementary Fig. 4a, b). FISH confirmed the presence of structural variants in *ABL1* or *BCL6*, which concur with the results of DNA-based NGS OncoPanel (Supplementary Fig. 4c). However, the loss of 5′ or 3′ region of *ABL1* or *BCL6* rather than creating fusion genes consisting of both 5′ and 3′ sides of the gene was observed. This is consistent with the fact that further RNA-seq did not support the expression of these ABL1 or BCL6 fusions.

Taken together, our analysis of the RNA-seq data suggested that while a variety of fusion events potentially occur, majority of the fusions detected by DNA-based sequencing did not lead to functional fusion oncogenes that mediate drug resistance.

### CRISPR-modified in vitro models harboring *EGFR* mutation and oncogenic fusions

**Fig. 5:**
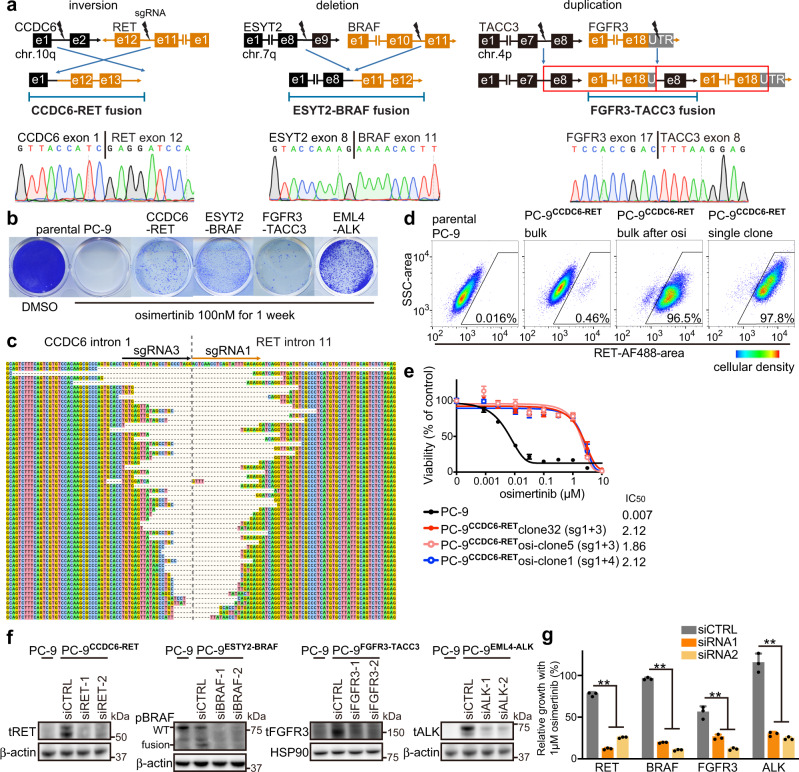
CRISPR-modified in vitro models, with *EGFR* mutation and oncogenic fusions. **a** The structures of the fusion oncogene and the location of designed single guide RNAs (sgRNAs) are shown, with representative sequencing chromatograms of fusion cDNA derived from bulk CRISPR-modified *EGFR* mutant PC-9 cells. e: exon; UTR: untranslated region. **b** Colony formation assay after 1 week of treatment with osimertinib, using parental PC-9 cells or CRISPR-modified PC-9 cells that express fusion oncogenes. **c** Breakpoints of *CCDC6-RET* fusion in bulk CRISPR-modified PC-9 cells. **d** Expression of RET protein in permeabilized parental or CRISPR-modified PC-9 models, evaluated with use of flowcytometry. PC-9^CCDC6-RET^ bulk cells were selected with 100 nM osimertinib for 1 week, and then a single clone was picked. Ratio of RET-expressing cells in each of four categories are indicated. Pseudo-color represents cellular density. **e** Results of cell viability assay after 72 h of osimertinib treatment. Half maximal inhibitory concentrations (IC50s) are shown for parental PC-9 cells and for single clones from CRISPR-modified PC-9^CCDC6-RET^ models, selected with or without 1 week of exposure to 100 nM osimertinib (*n* = 3 biological replicates, mean ± s.d.). **f** Knockdown of *RET*, *BRAF*, *FGFR3*, or *ALK* genes in CRISPR-modified PC-9 clones after 48 hours of siRNA treatment, is shown by western blot analyses. WT: wild type. **g** Knockdown of *RET*, *BRAF*, *FGFR3*, or *ALK* genes by siRNA in CRISPR-modified PC-9 cells resensitized them to 1 μM osimertinib (*n* = 3 biological replicates, mean ± s.d., two-sided *t* test, **p < 0.01). Source data of **e**–**g** are provided as a Source Data file.

To confirm the functional role of putative fusions in the context of drug resistance, we developed an in vitro system using *EGFR* del19 mutant lung cancer cell line PC-9 and CRISPR-Cas9 technology. Four different oncogenic fusions detected in our patients^8^: *CCDC6-RET*, *ESYT2-BRAF*, *FGFR3-TACC3*, and *EML4-ALK* were created (Fig. 5a and Supplementary Fig. 5a). These fusions can be classified as an inversion, deletion, and duplication based on their structure. *FGFR3-TACC3* was selected because this fusion was detected in 2 cases and consists of structural duplication.

In our patient with *FGFR3-TACC3*, the breakpoint was located in the untranslated region (UTR) of *FGFR3* exon 18 (4:1809119, 4:1737404)^8^. However, the patient-derived xenograft model from this patient (DFCI 361) displayed the presence of alternative splicing, which skipped exon 18 of *FGFR3* with a stop codon, and fused exon 17 of *FGFR3* with exon 8 of *TACC3* (Supplementary Fig. 5b). In PC-9 models (PC-9^FGFR3-TACC3^) edited by single guide RNAs (sgRNAs) targeting exon 18 UTR of *FGFR3*, we detected three kinds of splicing isoforms: two of these (isoforms #1 and #2) had a stop codon before fusion breakpoints, and thus could yield only FGFR3 protein but no fusion protein, while the third isoform skipped exon 18 of *FGFR3* (Supplementary Fig. 5b, c). sgRNA that targeted intron 17 of *FGFR3* efficiently yielded resistance-imparting cells selected by a short-term exposure to osimertinib compared with sgRNA targeting exon 18 UTR (Supplementary Fig. 5d).

Resistant colonies derived from PC-9 models edited for *RET*, *BRAF*, *FGFR3*, or *ALK* fusions grew under osimertinib treatment (Fig. 5b). Bulk PC-9^CCDC6-RET^ cells revealed a wide variety of fusion breakpoints between intron 1 of *CCDC6* and intron 11 of *RET* (Fig. 5c). Osimertinib treatment led to a selective increase in cells that expressed RET protein, as evaluated by flow cytometry (Fig. 5d and Supplementary Fig. 5e). Single cell clones that harbored *CCDC6-RET* or *ESYT2-BRAF* were obtained with or without osimertinib selection, and both showed resistance to osimertinib (Fig. 5e and Supplementary Fig. 5f). Use of siRNA to knockdown *RET*, *BRAF*, *FGFR3*, or *ALK* genes resensitized PC-9 models to osimertinib, thereby corroborating the functional impact of these fusions on osimertinib resistance (Fig. 5f, g).

To validate the putative *DLG1-BRAF* fusion detected by both OncoPanel and RNA-seq (Figs. 2 and 4b), we created *DLG1-BRAF* fusion in PC-9 cells and demonstrated that it causes resistance to osimertinib, while knockdown of *BRAF* by siRNA resensitized the cells to osimertinib (Supplementary Fig. 5g–i).

### Effectiveness of combination therapy evaluated by in vitro fusion models

**Fig. 6:**
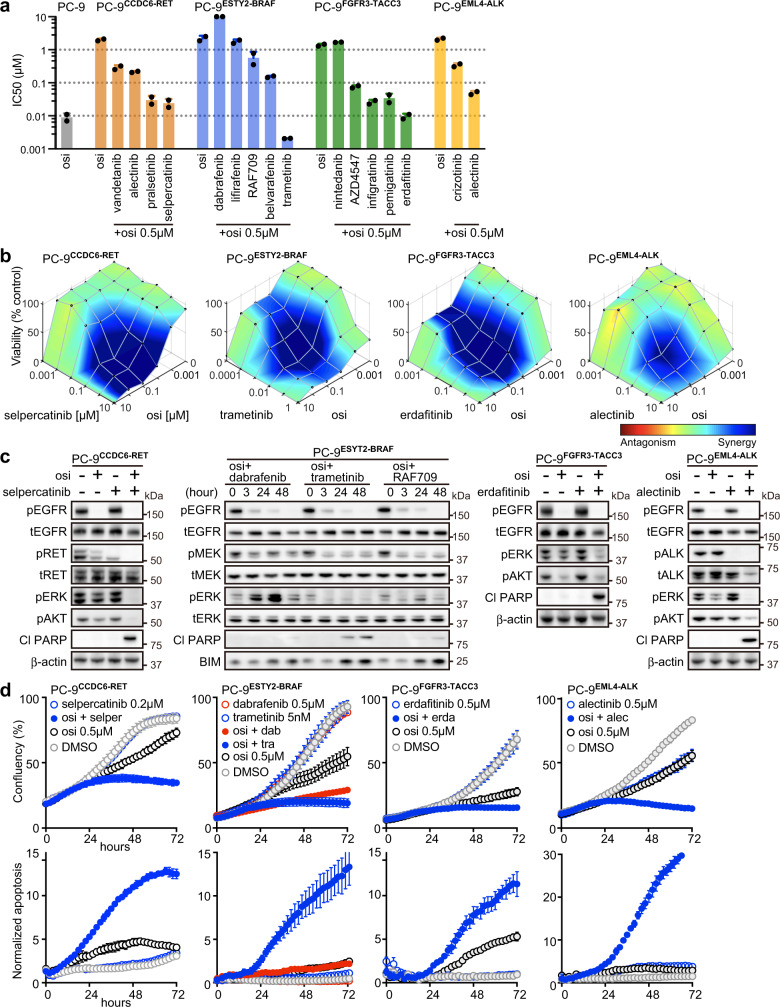
Effective combination therapies in CRISPR-modified fusion models. **a** The half maximal inhibitory concentrations (IC50s) of osimertinib and indicated drugs in parental PC-9 and in single clones from CRISPR-modified PC-9 models, after 72 h of treatment. For each fusion model, we used two single clones. Each dot indicates the mean of biological triplicate data. **b** Synergistic inhibitory effects of osimertinib and individual drugs in CRISPR-modified PC-9 models as shown by Combenefit (*n* = 2 biological replicates, mean). Pseudo-color represents synergy effects. **c** Western blot analyses following 48 h of treatment with 10 nM trametinib or 0.5 μM of other drugs, as indicated. **d** Proliferation of cells (top) and induction of caspase-3/7 (bottom) evaluated by Incucyte live-cell imaging (*n* = 3 biological replicates, mean ± s.d.). Source data of **a**–**d** are provided as a Source Data file.

To study and evaluate potentially effective therapies to be used in combination with osimertinib for cancers harboring fusion oncogenes, we screened drugs that targeted aberrantly expressing fusion oncogenes (Fig. 6a, b). Highly selective inhibitors such as pralsetinib, selpercatinib^25^, erdafitinib, and alectinib were more effective than multi-kinase inhibitors (Fig. 6a). Of note, persistent ERK1/2 activation in PC-9 ^ESYT2-BRAF^ cells was detected following treatment with osimertinib plus RAF inhibitors dabrafenib or RAF709, but not following osimertinib plus MEK inhibitor trametinib (Fig. 6c). Combination therapies targeting mutant *EGFR* and the acquired oncogenic fusion partners induced growth inhibition and apoptosis over time (Fig. 6d).

### Mechanisms of acquired resistance to combination therapy

**Fig. 7:**
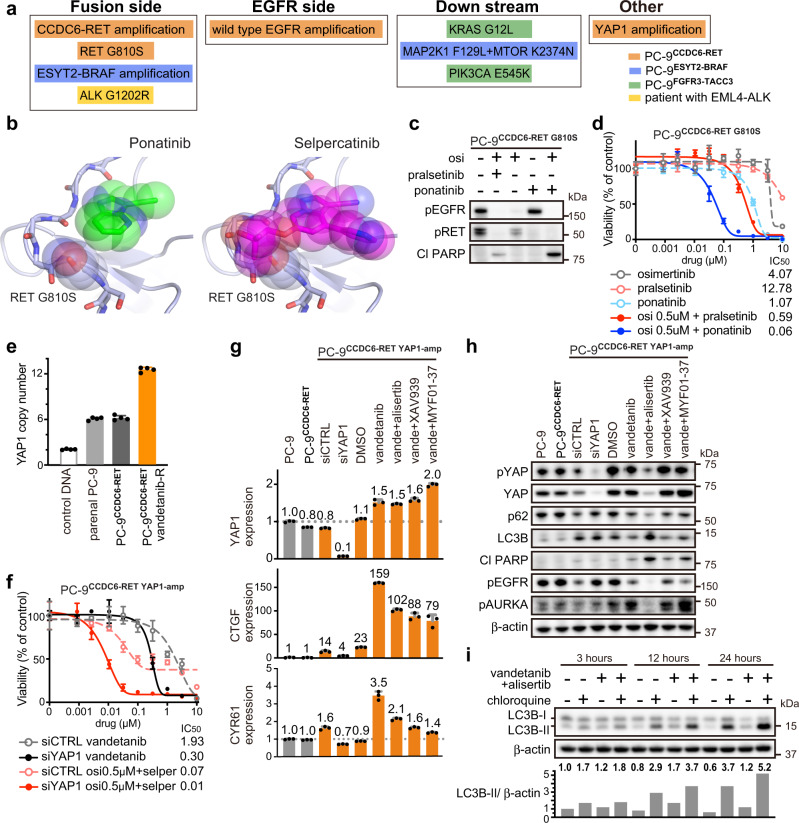
Mechanisms of acquired resistance to combination therapy in a patient and in in vitro models. **a** Summary of mechanisms of acquired resistance to inhibition of *EGFR* and fusion genes in a patient, as well as in CRISPR-modified PC-9 models. osi: osimertinib. **b** Crystal structure of RET in a complex with ponatinib or selpercatinib. Modeling the G810S mutation shows the resulting clashes with selpercatinib, but not with ponatinib. **c** Western blot analyses following 48 h of treatment with 0.5 μM of the indicated drugs in PC-9^CCDC6-RET^ models that had acquired *RET* G810S mutation, after being exposed to osimertinib plus pralsetinib. **d** Cell viability assay after 72 h of treatment of PC-9 model harboring *RET* G810S mutation (*n* = 3 biological replicates, mean ± s.d.). **e** Copy number of *YAP1* evaluated by quantitative PCR. *RNaseP* was used as an internal control (*n* = 4 biological replicates, mean ± s.d.). **f** Cell viability assay following 72 h of treatment with indicated drugs in PC-9 model harboring *YAP1* amplification (*n* = 3 biological replicates, mean ± s.d.). **g** Expression of *YAP1*, *CTGF*, and *CYR61* evaluated by qPCR; *GUSB* was used as an internal control. PC-9 models were treated for 48 hours with siRNA, 10 μM of MYF01-37, or 1 μM of other indicated drugs (*n* = 3 biological replicates, mean ± s.d.). **h** Western blot analyses following 48 h of treatment with siRNA, 10 μM of MYF01-37, or 1 μM of other drugs, as indicated. **i** Results of Autophagy Flux Assay, using 15 μM of chloroquine or 1 μM of indicated drugs in PC-9 model with *YAP1* amplification. Ratios of LC3B-II to β-actin were quantified by Image J software (*n* = 1). Source data of **c**–**i** are provided as a Source Data file.

Although mechanisms of resistance to single agent therapies are well described, limited information exists on resistance mechanisms to combination treatment. To uncover the resistance mechanisms to combination therapy, we established resistant models from PC-9^CCDC6-RET^, PC-9^ESYT2-BRAF^, and PC-9^FGFR3-TACC3^ by chronically exposing each cell line to a combination of EGFR inhibitors and inhibitors of RET, BRAF, or FGFR. These models revealed a wide variety of resistance mechanisms including amplification of the fusion oncogene, secondary mutations and/or amplification in genes critical for downstream signaling (Fig. 7a and Supplementary Table 4).

Resistance to the EGFR and RET inhibitor combination was particularly illustrative. Amplification of *CCDC6-RET*, a resistance mechanism to the combination of alectinib plus osimertinib detected by qPCR, was overcome by more potent and selective RET inhibitors pralsetinib or selpercatinib (Supplementary Fig. 6a–c). Further exposure to pralsetinib induced *RET* G810S mutation, which demonstrated cross-resistance to selpercatinib (Supplementary Fig. 6d, e). Drug screening revealed that ponatinib, an FDA-approved multi-kinase inhibitor for leukemia, was effective against *RET* G810S, which was confirmed by analyses of the crystal structure of RET using computer-aided docking poses of truncated analogs of selpercatinib and ponatinib: the resulting serine alcohol in *RET* G810S clashes with selpercatinib but not with ponatinib (Fig. 7b–d and Supplementary Fig. 6f). Amplification of wild type *EGFR*, induced by selpercatinib plus osimertinib (*EGFR* mutant-selective inhibitor) and detected by qPCR, could be overcome by EGFR inhibitors afatinib or dacomitinib, which inhibit both mutant and wild-type EGFR (Supplementary Fig. 6g, h). The time to development of in vitro resistance was shorter in models that developed an amplification as the resistance mechanism compared to the model that developed the *RET* G810S mutation (Supplementary Table 5).

Additionally, amplification of *YAP1* was detected by NGS and qPCR in PC-9^CCDC6-RET^ exposed to vandetanib, which inhibits both EGFR and RET (Fig. 7e). Knockdown of *YAP1* by siRNA resensitized cells to vandetanib (and osimertinib plus selpercatinib), confirming the role of *YAP1* amplification as a resistance mechanism (Fig. 7f). Although the TEAD inhibitor MYF01-37 or tankyrase inhibitor XAV939 were effective in inhibiting YAP1 in our previous study^26^, current *YAP1*-amplified models were resistant to these drugs (Supplementary Fig. 6i). Phospho-receptor tyrosine kinase array revealed elevated expression of pMET only in the presence of vandetanib, which was confirmed by western blotting despite a lack of *MET* mutations or amplifications (Supplementary Fig. 6j, k). This upregulation of pMET suggested an epigenetic mechanism and led us to screen drugs related to epigenetic modulators such as aurora kinase (AURK), bromodomain and extra-terminal motif (BET), as well as protein kinase C (PKC) inhibitors. Combination of AURK inhibitors and vandetanib had a synergistic effect (Supplementary Fig. 6l, m). To clarify the underlying mechanisms of the discrepancy that the *YAP1*-amplified model responded only to AURK inhibitors but not to MYF01-37 nor XAV939, we evaluated the expression of *YAP1* and YAP activity, as measured by expression of its known downstream target genes *CTGF* and C*YR61*. YAP activity (specifically CTGF) increased dramatically following acquisition of *YAP1* amplification, but the extent of inhibition of *YAP1*, *CTGF*, and *CYR61* was similar among MYF01-37, XAV939, and alisertib (Fig. 7g). We thus focused next on post-transcriptional protein levels. The AURK inhibitor alisertib, but not MYF01-37 or XAV939, reduced protein levels of YAP as well as p62, and increased those of LC3B (p62 is the cargo receptor protein that interacts with autophagic substrates for autophagosomic-lysosomal degradation, while LC3B is an autophagosome marker) (Fig. 7h). Induction of autophagy was confirmed by the Autophagy Flux Assay, which evaluates the conversion of LC3B-I to LC3B-II through lipidation (Fig. 7i). Taken together, these findings suggest that *YAP1* amplification can be overcome through inhibition of YAP activity itself and induction of autophagy by the combined use of vandetanib and AURK inhibitors.

In PC-9^ESYT2-BRAF^ cells, amplification of *ESYT2-BRAF* was detected by qPCR after exposure to osimertinib and trametinib (Supplementary Fig. 7a, b). Interestingly, knockdown of *BRAF* by siRNA resensitized these cells to trametinib alone without the requirement for concomitant EGFR inhibition (Supplementary Fig. 7c, d). The RAF inhibitor RAF709, which inhibits dimerized BRAF^27^, showed a synergistic effect with trametinib thus phenocopying the siRNA results (Supplementary Fig. 7e, f). Further exposure to trametinib plus RAF709 induced concurrent *MAP2K1* F129L and *MTOR* K2374N mutations in 9 out of 10 single clones, which could be overcome by the combination of ERK inhibitor SCH772984 and mTOR inhibitor everolimus (Supplementary Fig. 7g–i).

Finally, the PC-9^FGFR3-TACC3^ model revealed *KRAS* G12L or *PIK3CA* E545K mutations after exposure to osimertinib plus erdafitinib or AZD4547 – and these resistance mechanisms could be overcome by adding trametinib or PI3K inhibitor alpelisib, respectively (Supplementary Fig. 8a–d).

## Discussion

Mechanisms of acquired resistance to EGFR inhibitors in *EGFR* mutant lung cancer are diverse and include genomic mechanisms such as point mutations, amplifications, and oncogenic fusions. Of these, oncogenic fusions are the most difficult to detect using targeted NGS and the least well studied comprehensively. In the current manuscript, we systematically evaluate the functional significance of putative fusion oncogenes as drug resistance mechanisms in *EGFR* mutant cancers using both preclinical models and patients treated with single agent EGFR inhibitors and/or combination therapies. We demonstrate that both functional and non-functional fusion oncogenes in terms of drug resistance are present in *EGFR* mutant cancers as detected by DNA-based NGS. By performing RNA-based studies and functional in vitro studies, we were able to identify which of the putative fusion oncogenes were indeed true mediators of EGFR inhibitor resistance. These findings are important to recognize by treating physicians, since rearrangements, even in putative oncogenes, do not always result in functional alterations leading to clinical drug resistance.

Intriguingly, our studies reveal that *RET*, *ABL1*, and *NRG1* fusions with uncertain or uncommon partner genes in fact have the same common genomic breakpoints as those that form functional *CCDC6-RET*^28^, *BCR-ABL1*^29^, or CD74-*NRG1* fusions^30^. However, our validation results showed that fusions involving these uncertain partners did not lead to drug resistance. Although there is a significant bias in favor of detecting fusions with these common breakpoints because OncoPanel assay was designed to capture clinically relevant fusions, these findings suggest that, in the evolution of oncogenic fusions, multiple fusions can occur at specific fragile regions in cancer cells with increasing genomic instability^31^. These fusions in turn can lead to cell death, no change of gene expression, or can induce gene expression of oncogenes. Once the fusion is created with specific partner genes, which enables activation of the original oncogene, the oncogenic fusion is subsequently selected under drug pressure. These findings are further supported by recent reports on the major *ALK*, *ROS1*, and *RET* fusions in which uncommon breakpoints may indicate a non-functional fusion^32^ and a shorter clinical response^33^ relative to that in fusions with common breakpoints. It remains unclear if these fusions with uncommon breakpoints potentially have a functional role other than drug resistance.

Our CRISPR models using *EGFR* mutant lung cancer cell lines, enabled precise evaluation of the biology and drug resistance as a result of a concurrent fusion oncogene which would not have been possible using conventional simple Ba/F3 models. Our models allow the evaluation of downstream signaling imparted by fusion oncogenes, whose expression is driven by the endogenous promoter, in the context of bona fide lung cancer cell lines. Additionally, these models are also useful for evaluating splicing events and for validating the oncogenic function of putative fusions, regardless of the length of the coding sequences, whereas viral vectors are limited by the insert size^15^. As combination treatments are being used with increasing frequency, these models are also useful in modeling potential drug resistance mechanisms.

Combination targeted therapy-resistant cell lines are then useful for both understanding the biology of resistance and as models in which to test treatment strategies. As such, they can reveal both on-target and downstream resistance mechanisms, which are not feasible in Ba/F3 cells. This is exemplified by the identification of *RET* G810S, which has also been identified in patients^34^, as causing clinical drug resistance in the setting of RET oncogene fusions. Using the *EGFR* del19/*CCDC6-RET* G810S cell line, we were able to identify ponatinib as a potential treatment strategy and validate the osimertinib/ponatinib combination using this cell line model (Fig. 7b–d). Although the clinical efficacy of ponatinib was not promising in patients with *RET* fusion likely due to the off-target toxicity and insufficient concentration of the dug^35^, we showed a proof of concept that drugs which do not structurally interfere with the G810 solvent front mutation can overcome resistance. RET inhibitors including TPX-0046 are in development in clinical trials (NCT04161391). We also identified *YAP1* amplification as a mechanism of resistance to RET inhibition and revealed a mechanistic basis of how alisertib can overcome this mechanism of resistance. Interestingly, resistance mediated by *YAP1* amplification could not be reversed by tankyrase inhibition (indirect inhibitor of YAP) or by a TEAD inhibitor which have been effective in blocking drug induced YAP activity (Supplementary Fig. 6i)^26^. These findings suggest that the mechanism of YAP activation (amplification vs. increased activity) may dictate the therapeutic approach necessary to combat drug resistance. We further observed amplification of *CCDC6-RET* (in cells treated with alectinib and osimertinib) and wild type *EGFR* (in cells treated with selpercatinib and osimertinib). Intriguingly, given that alectinib is a less potent RET inhibitor compared to selpercatinib and osimertinib does not inhibit wild type EGFR as well as mutant EGFR, these findings suggest that amplification of *RET* and wild-type *EGFR*, respectively are involved in the rapid development of drug resistance. Amplification of *CCDC6-RET* and wild type *EGFR* may represent easier and faster routes to the development of resistance compared to the selection of clone harboring a secondary drug resistance mutation. This hypothesis concurs with data on the time to resistance in these models (Supplementary Table 5) but will require additional clinical data on patients treated with combination therapies.

The limitations of this study include the following: only oncogene-related fusions were studied, a subset of samples was validated by RNA-seq, not all fusion partners were identified which might be detectable by further genomic analyses.

In summary, our genomic and functional studies of fusion oncogenes as potential drug resistance mechanisms to EGFR inhibitors provides insight into the biological complexity of fusion oncogenes. Currently, no single assay is adequate for detecting unknown but functional fusion oncogenes. In clinical practice, we propose starting with DNA-based NGS which can capture certain well-described fusions as well as other known resistance mechanisms including amplifications and point mutations. Then, RNA-based anchored multiplex PCR for targeted NGS^36^ could detect further atypical but actionable fusions although the careful evaluation of raw reads is needed, exemplified by our case with *GKAP1-NTRK2*. Finally, in collaboration with research laboratories, bulk RNA sequencing and in vitro modeling could further validate the biological significance of unknown fusions^15^. Understanding the complex biology of fusion genes is vital for developing further assays including those in the liquid biopsy field, where fusion detection is more challenging^37^, clinical indications, and for leveraging effective combination therapy to overcome drug resistance.

## Methods

### Study protocol

DFCI IRB and the Hospital Sírio-Libanês IRB approved the protocol for this study. Patients provided written informed consent according to CARE guidelines and in compliance with the Declaration of Helsinki principles.

### Cell lines and drugs

The PC-9 NSCLC cell line harboring *EGFR* mutant (del E746_A750) was originally established in Tokyo Medical University and obtained from Dr. Nishio Kazuto (Kindai University, Osaka, Japan) in 2005, and confirmed by fingerprinting. PC-9 cells were grown in RPMI-1640 (Gibco), 10% FBS, and 1% penicillin/streptomycin (Gibco). Throughout the study, cells were periodically tested for Mycoplasma, using the Mycoplasma Plus PCR Primer Set (Agilent). All drugs used are listed in Supplementary Data 1.

### Genome editing with use of CRISPR-Cas9

To create fusion genes in PC-9 cell lines, sgRNAs were designed using Deskgen (deskgen.com), based on the proximity to the patient’s breakpoints and the off-target effects. crRNAs (Integrated DNA Technologies, IDT) were hybridized with tracrRNAs to make 150 pmol sgRNAs, and the ribonucleoprotein complex was formed in vitro with 120 pmol Cas9 Nuclease (IDT). Reaction mixtures were nucleofected into PC-9 cells (1 × 10^5^ cells), and suspended in 20 µl of SE solution (Lonza) using Lonza 4D-Nucleofector (Lonza) with EN-138 mode. DNA was extracted from single clones using the QuickExtract DNA Extraction Solution (Licigen). RNA was extracted from bulk cells using the RNeasy Mini kit (Qiagen) and cDNA was synthesized using the QuantiTect Reverse Transcription Kit (Qiagen). Fusions were confirmed by Sanger sequencing (Genewiz) or by CRISPR sequencing at the DNA sequencing core at Massachusetts General Hospital (MGH). All sgRNAs and primers are listed in Supplementary Data 1.

### Colony formation assay

Bulk PC-9 cells, edited to contain fusions (1 × 10^5^ cells), were seeded into 12-well plates and cultured with or without 30 nM osimertinib. After staining with 0.5% crystal violet in 25% methanol for 30 min, cells were imaged on a scanner.

### Intracellular staining for RET and flow cytometry

Cells (3 × 10^5^) were washed in PBS + 10% FBS, fixed for 10 min at room temperature in 2% paraformaldehyde, washed in PBS + 10% FBS, and permeabilized in cold 90% methanol for 30 min on ice; they were then washed and incubated for 1 h at room temperature in an anti-RET antibody solution (1:50) in PBS + 10% FBS, washed and stained with anti-rabbit Alexa Fluor 488 secondary antibody (1:500; 30 min, room temperature). Cells were washed, resuspended in PBS + 10% FBS, and analyzed on a BD LSR Fortessa flow cytometer (BD Biosciences). Data were shown using FlowJo v10 (FlowJo, LLC).

### Gene knock-down by siRNA

Control siRNA or target-specific siRNA (final concentration of 10 nM, Life Technologies) and Lipofectamine RNAiMAX Transfection Reagent (final concentration of 0.3%, Thermo Fisher) were mixed in Opti-MEM (Gibco) for 10 minutes, then added to the growth medium of CRISPR-modified PC-9 cell lines. For the growth inhibition assay, cells were trypsinized 24 h after transfection, cultured in 384-well plates for 24 h, and treated with drugs. For western blot or qPCR analyses, samples were collected 48 h after transfection.

### Cell growth inhibition assay

Parental or CRISPR-modified PC-9 cell lines (1 × 10^3^ cells) were plated in 384-well plates. After 24 h, cells were treated for 72 h with drugs at the indicated concentrations. Endpoint cell viability assays were performed using Cell Titer Glo (Promega). Bliss drug synergy was calculated using the Combenefit software version 2.021^38^.

### IncuCyte assays

Cells were plated into 96-well plates (1 × 10^3^ cells/well) in 200 µl of growth medium; drugs were added the next day. Confluency was measured every 2 h, using the IncuCyteS3 Live-Cell Imaging Analysis System (Essen Bioscience). For apoptosis studies, cells were treated with inhibitors added to media containing the CellEvent Caspase 3/7 Green ReadyProbes reagent (Thermo Fisher Scientific)^26^.

### Quantitative RT-PCR

qPCR reactions were set up in 20 µl, using the TaqMan Gene Expression Master Mix (Thermo Fisher), including 1 µl of 1:5 diluted cDNA synthesized from 1 µg RNA extracted with RNeasy Plus Mini Kit (QIAGEN). Reactions were run in the StepOne Plus Real-time PCR System (Applied Biosystems). For each sample, expression levels of the target genes were normalized to those of the GUSB housekeeping gene. Primers and probes are listed in Supplementary Data 1.

### Gene copy number analyses by qPCR

qPCR reactions were set up in 20 µl, using the TaqPath ProAmp Master Mix (Thermo Fisher), including 20 ng of DNA extracted with DNeasy Blood & Tissue Kit (QIAGEN). Reactions were run in the StepOne Plus Real-time PCR System (Applied Biosystems). Copy numbers of target genes were normalized to those of the RNaseP housekeeping gene in each sample, along with a control human genomic DNA (Promega). Primers and probes are listed in Supplementary Data 1.

### Western blot analysis

Cells were lysed with RIPA buffer (Boston Bioproducts) supplemented with a cOmplete Mini EDTA-free Protease inhibitor cocktail (Roche) and a PhoSTOP phosphatase inhibitor cocktail (Roche). The total cell lysate (20 μg) was subjected to SDS polyacrylamide gel electrophoresis and transferred to Immobilon-P polyvinylidene difluoride membranes (Bio-Rad Laboratories). Antibodies used are listed in Supplementary Data 1.

### Phospho-receptor tyrosine kinase (RTK) array analysis

A Human Phospho-RTK Array Kit (R&D Systems) was used to measure the relative levels of tyrosine phosphorylation of 42 RTKs. Cells were lysed and 200 µg of each lysate was incubated with antibody, according to the manufacturer’s instructions.

### Autophagy flux assay

PC-9 cells with *YAP1* amplification were treated with 1 µM of the indicated drug, with or without 15 µM of chloroquine^39^. Protein lysates were collected as described above. The ratio of LC3B-II/ β-actin was calculated with use of Image J software.

### Simulation of structural docking

A docking model of the RET kinase domain was constructed from a docking grid, utilizing coordinates from a co-crystal structure of RET in complex with nintedanib (PDB ID 6NEC). Truncated models of ponatinib and loxo292 were prepared with LigPrep, and docked with RET, using GLIDE (Schrödinger, Inc). The G810S point mutation was generated from the docking model with use of PyMOL (Schrödinger, Inc.)

### Clinical data at Dana-Farber Cancer Institute

Using an IRB-approved protocol with written informed consent, a pan-cancer cohort (*n* = 22,742) analyzed by DNA-based hybrid capture NGS OncoPanel at the Dana-Farber Cancer Institute/Brigham and Women’s hospital^17,40,41^ was queried, to find patients with NSCLCs that harbored a sensitizing *EGFR* mutation as well as fusions.

OncoPanel is a cancer genomic assay to detect somatic mutations, copy number variations, and structural variants in tumor DNA extracted from fresh, frozen, or FFPE. The OncoPanel assay surveys exonic DNA sequences of 447 cancer genes and 191 regions across 60 genes for rearrangement detection. DNA was isolated from tissue containing at least 20% tumor nuclei using QIAamp DNA mini kit (QIAGEN). DNA was quantified with PicoGreen (ThermoFisher), and 200 ng of DNA was used for library preparation (with a low input threshold of 50 ng). Hybrid-capture libraries were prepared using SureSelect hybrid capture kit (Agilent). Sequencing was performed using an Illumina HiSeq 2500 with 2×100 paired- end reads to a mean target coverage of 187X unique, high quality, mapped reads per sample (range 50 to 844X; 50X minimum required to pass). Sequence reads were aligned to the reference sequence b37 edition from the Human Genome Reference Consortium by using bwa and further processed with Picard (version 1.90; http://broadinstitute.github.io/picard/) to remove duplicates and with Genome Analysis Toolkit (GATK, version 1.6-5-g557da77) to perform localized realignment around insertion and deletion (indel) sites. Copy number variants were called with the internally developed algorithms RobustCNV.

Fusions from OncoPanel data were detected using BreaKmer, which identifies structural variants via realignment of contigs formed by assembly of aberrant reads (soft-clipped alignments and unmapped reads with mapped mates) in targeted regions^42^. To focus on the fusions consisting of two different genes, intragenic fusions including small deletion in the same gene were excluded from this study. The fusions were classified into five groups: unknown oncogenes that have not been reported in lung cancer, lung cancer oncogenes, tumor suppressor genes, genes of unknown significance, or amplifications based on the copy number of 5′ or 3′ genes. For each patient, the clinical history and duration of treatment with EGFR-TKIs was collected by manual chart review. OncoPanel data for each patient was analyzed, to clarify whether or not unknown fusions were present after acquiring TKI resistance. In one case with *EML4-ALK*, confirmatory immunohistochemistry was done, using an antibody against ALK (clone 5A4; Leica Biosystems) or against EGFR-L858R (clone 43B2; Cell Signaling). Fluorescence in-situ hybridization (FISH) was done using Vysis LSI ALK Dual color, Break Apart Rearrangement Probe (Abbott Molecular), Vysis LSI BCL6 Dual color, Break Apart Rearrangement Probe, and ABL1 Break Apart (vB) FISH Probe (Empire Genomics).

### RNA-based targeted NGS in Hospital Sírio-Libanês

RNA extracted from FFPE samples using ReliaPrep RNA Miniprep Systems (Promega) from Case #4 was analyzed using the hybrid capture panel Illumina TruSight™Oncology 500 (TSO500). RNA analysis covers fusions in 55 genes and oncogenic isoforms/alternative splicing variants in three genes. NGS applies the Illumina NextSeq 550. TSO500 uses the software Illumina TSO500 Local App 2.0.1.4, along with a personalized analysis pipeline in the Clinical Genomics Workspace platform of PierianDx.

### RNA sequencing

Using the same IRB-approved protocol listed above, we sent archived clinical samples – including FFPE tissue and leftover RNA from prior clinical genomic sequencing tests – to the Broad Institute for Transcriptome Capture or Whole Transcriptome assays, respectively. Given that FFPE samples are not the best material to perform RNA-seq, we applied Transcriptome Capture assay which is optimized for FFPE samples.

For the Transcriptome Capture assay, RNA was extracted using AllPrep DNA/RNA FFPE Kit (QIAGEN) and RiboGreen quantified and assessed for quality using a LapChip GX RNA Caliper system. The threshold of the quality of samples are as follows: >550 ng total RNA, >12 ng/μl concentration, RNA Quality Score [RQS] > 5.5, DV200 > 0.3. Total RNA was normalized to 5 ng/μl. In all, 2 μL of ERCC controls were spiked into each sample as well as a k562 control. An aliquot of 200 ng for each sample was taken for library preparation using Illumina TruSeq™ Stranded mRNA Sample Preparation Kit. The resultant 400 bp cDNA then went through dual-indexed library preparation. After normalizing samples to 5 ng/μL, the set was pooled and quantified using the KAPA Library Quantification Kit for Illumina Sequencing Platforms. The entire process was done by either Agilent Bravo or Hamilton Starlet. Pooled libraries were normalized to 2 nM and denatured using 0.1 N NaOH prior to sequencing. Flowcell cluster amplification and sequencing were performed according to the manufacturer’s protocols using the NovaSeq. Each run was a 101 bp paired-end with an eight-base index barcode read. Data were analyzed using the Broad Picard Pipeline, which includes de-multiplexing and data aggregation. Alignment was completed using the STAR alignment algorithm against human reference hg19.

In Whole Transcriptome assay, the threshold of the quality of samples are as follows: >250 ng total RNA, >2 ng/μL concentration, RQS > 5.5. Total RNA was normalized to 5 ng/μl. Following plating, 2 μL of ERCC controls (using a 1:1000 dilution) were spiked into each sample. Using Illumina’s TruSeq RNA Access Library Prep kit, a stranded cDNA library was prepared from isolated RNA, which was then hybridized to a set of DNA oligonucleotide probes to enrich the library for mRNA transcript fragments. Flowcell cluster amplification and sequencing were performed according to the manufacturer’s protocols using NovaSeq. Each run was a 76 bp paired-end with an eight-base index barcode read. Data were analyzed using the Broad Picard Pipeline which includes de-multiplexing and data aggregation. Alignment was completed using the STAR alignment algorithm against human reference hg19. Transcriptome Capture covered the RefSeq and GENCODE v12 databases to >98%. In both assays, samples which pass the quality-check were processed with the goal of reaching 50 million reads aligned in pairs.

BAM files preprocessed by the Broad Institute were reverted to unmapped BAMs and converted to fastq files, using Picard (v1.11.5) functionalities. Candidate somatic fusions were called by three fusion callers, STAR Fusion (v1.2.0), FusionCatcher (v1.00) and ChimPipe (v0.9.6) with default parameters. STAR Fusion was run with the Trinity Cancer Transcriptome Analysis Toolkit (CTAT) genome library (vNov012017), FusionCatcher was run with FusionCatcher’s human database (v90), and ChimPipe was run with the GENCODE annotation library (v19). The candidate fusion calls indicated by each caller were normalized and consensus fusions were identified for each sample.

Discordant reads were identified using SAMtools (v0.1.19) using the “-F 1294” flag. A BED file of the discordant reads was created using the “bamtobed” function of BEDTools (v2.25.0), with the “-bedpe” argument included to facilitate further analysis of the discordant read pairs. To produce the BigWig files appropriate for visualizing in the IGV browser, read coverage was calculated using BAM files and the “bamCoverage” function of deepTools (v3.5.0) with default parameters.

### Statistics and reproducibility

Mean values were assessed using unpaired two-tailed Student’s *t* test. Columns in the figures represent means ± standard deviation. Asterisks used to indicate significance correspond to: *p < 0.05, **p < 0.01. GraphPad Prism9 was used for all statistical analyses. All experiments have been performed in at least two independent experiments.

### Reporting summary

Further information on research design is available in the Nature Research Reporting Summary linked to this article.

## Supplementary information

Supplementary Information

Dataset 1

Reporting Summary

## Data Availability

Raw fastq files of RNA-seq data from clinical samples generated in this study have been deposited in the NCBI GEO database under accession code GSE182323. Protein data were obtained from PDB ID 6NEC and 4U0I. Analyzed OncoPanel data in this manuscript were deposited into AACR GENIE project for public access [https://www.aacr.org/professionals/research/aacr-project-genie/aacr-project-genie-data/] but access to raw fastq files of OncoPanel is restricted to investigators approved by the DFCI IRB. Source data are provided with this paper.

## Source data

Source Data
