# Supplementary material for: Genomic and biological study of fusion genes as resistance mechanisms to EGFR inhibitors
Source: Nat Commun. 2022 Sep 24;13:5614. doi: 10.1038/s41467-022-33210-2 (PMC9509394; doi:10.1038/s41467-022-33210-2)
Supplement: Supplementary file 1 — Supplementary Information [file 41467_2022_33210_MOESM1_ESM.pdf]

Supplementary Fig. 1

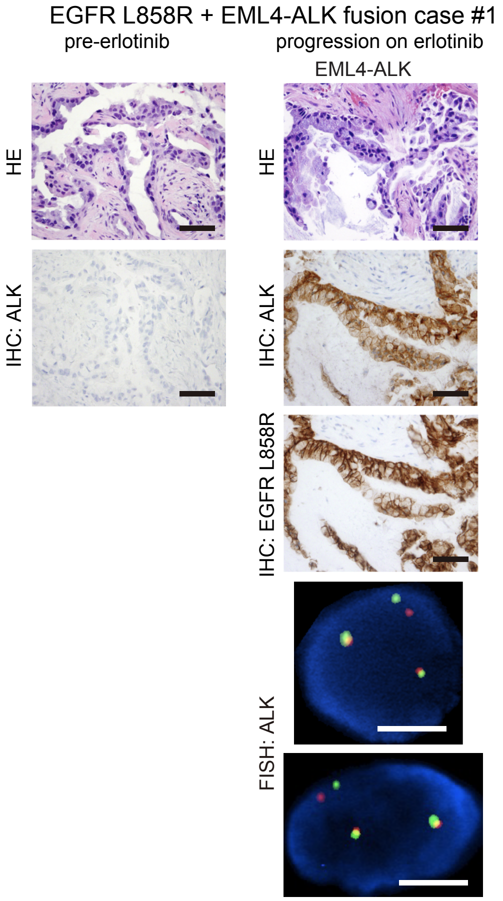

Supplementary Fig. 1. *EGFR* L858R mutant adenocarcinoma acquired *ALK* fusion

EGFR L858R mutant adenocarcinoma acquired *ALK* fusion, which was detected by *ALK* break-apart fluorescence in situ hybridization (FISH) – as exemplified by a split green (5' centromeric) signal and a red (3' telomeric) signal. Confirmatory immunohistochemistry (IHC) shows expression of ALK protein along with mutant-specific EGFR-L858R protein in the same tumor cells. HE: hematoxylin-eosin stain. Scale bars: black 100 µm, white 5 µm.

Supplementary Fig. 2

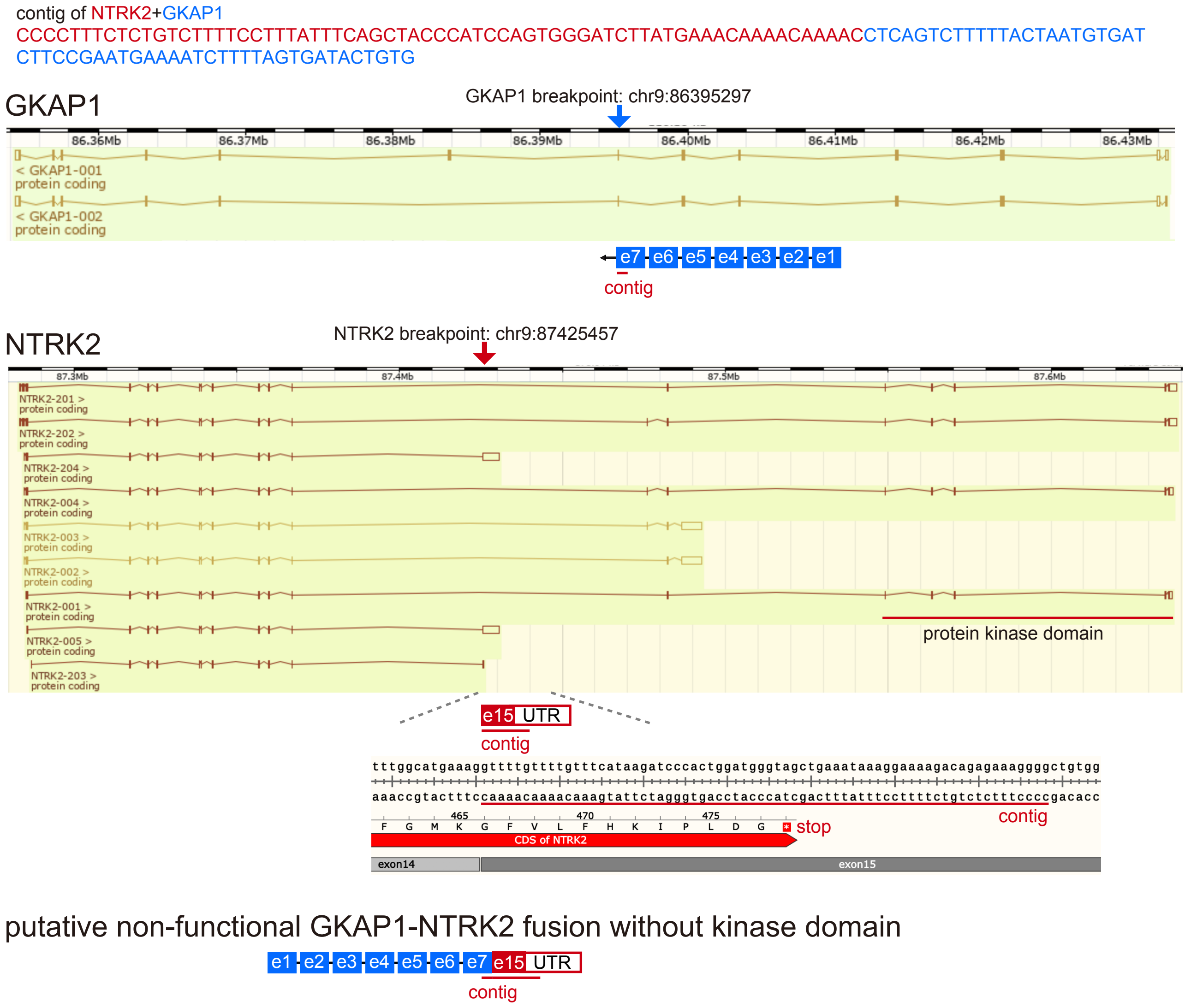

Supplementary Fig. 2. Putative *GKAP1-NTRK2* fusion detected by RNA-based targeted sequencing
Contig detected by hybrid capture RNA-based target sequencing (Illumina TruSight™Oncology 500) were shown in isoforms of *GKAP1* and *NTRK2;* these isoforms were obtained from the Ensemble Genome Browser (https://asia.ensembl.org/index.html). Contig was located at the end of *GKAP1* exon 7, and at the beginning of *NTRK2* exon15, including the stop codon and untranslated regions (UTRs). Thus, the putative *GKAP1-NTRK2* fusion does not include the kinase domain of *NTRK2*, and as such it cannot be functional.

Supplementary Fig. 3

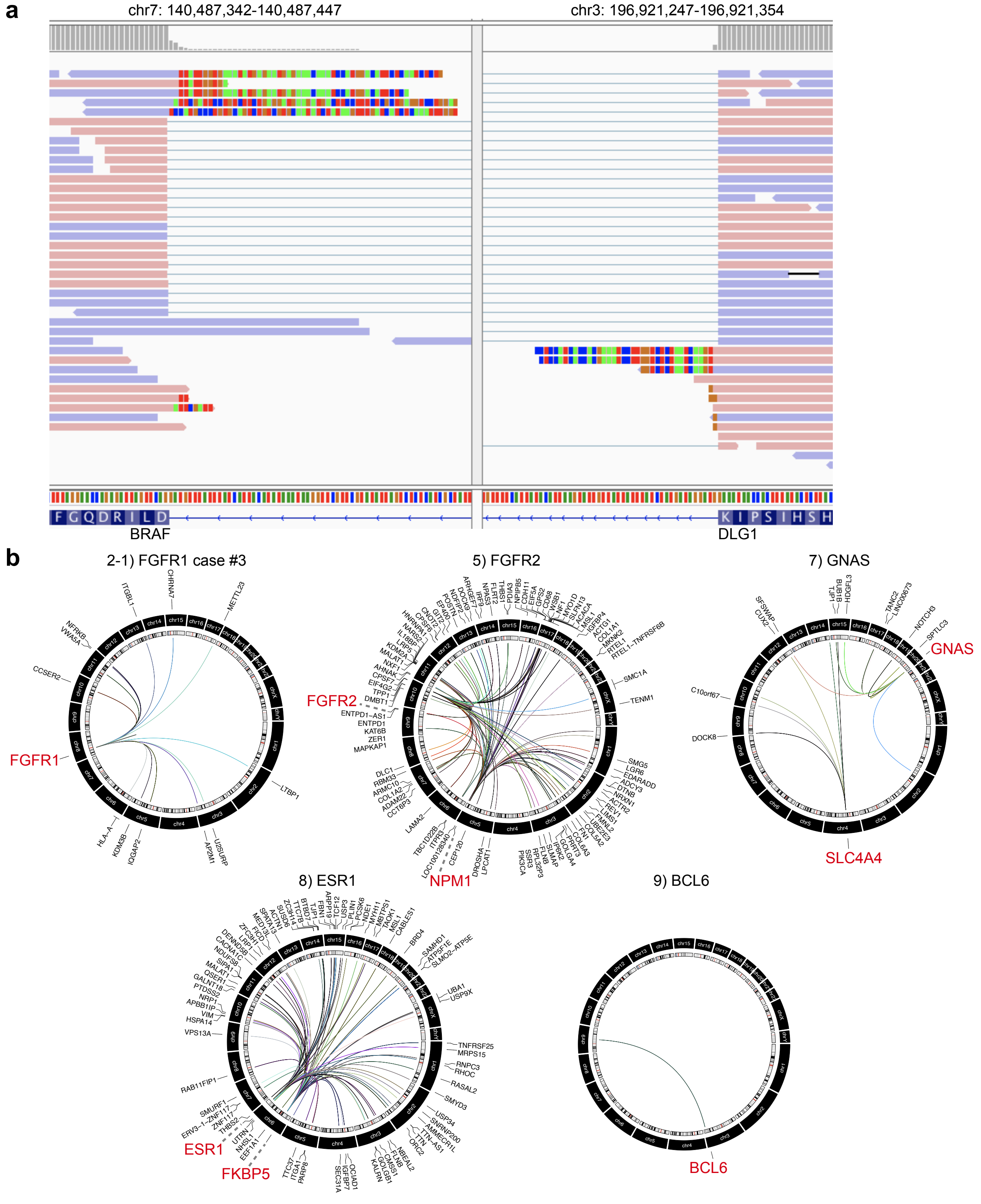

Supplementary Fig. 3. Discordant reads with putative fusions, detected by RNA-sequencing

(**a**) RNA-sequencing data showing *DLG1-BRAF* fusion visualized using IGV browser. The reads at both fusion breakpoints are presented with the direction of the reads indicated by the pink or purple colors. The rainbow colors at the ends of some reads represent the clipped region of that read, indicating bases that do not align to the original positions. The fusion callers use these clipped reads to identify the fusion partner by locating the position in the genome where the clipped region aligns best. (**b**) Circos plots showing discordant reads with *FGFR1, NPM1, FGFR2, SLC4A4, GNAS, FKBP5, ESR1, and BCL6,* detected via RNA sequencing. Samples are derived from patients with *EGFR* mutant lung cancer in which putative fusions were detected by DNA-based next generation sequencing OncoPanel.

Supplementary Fig. 4

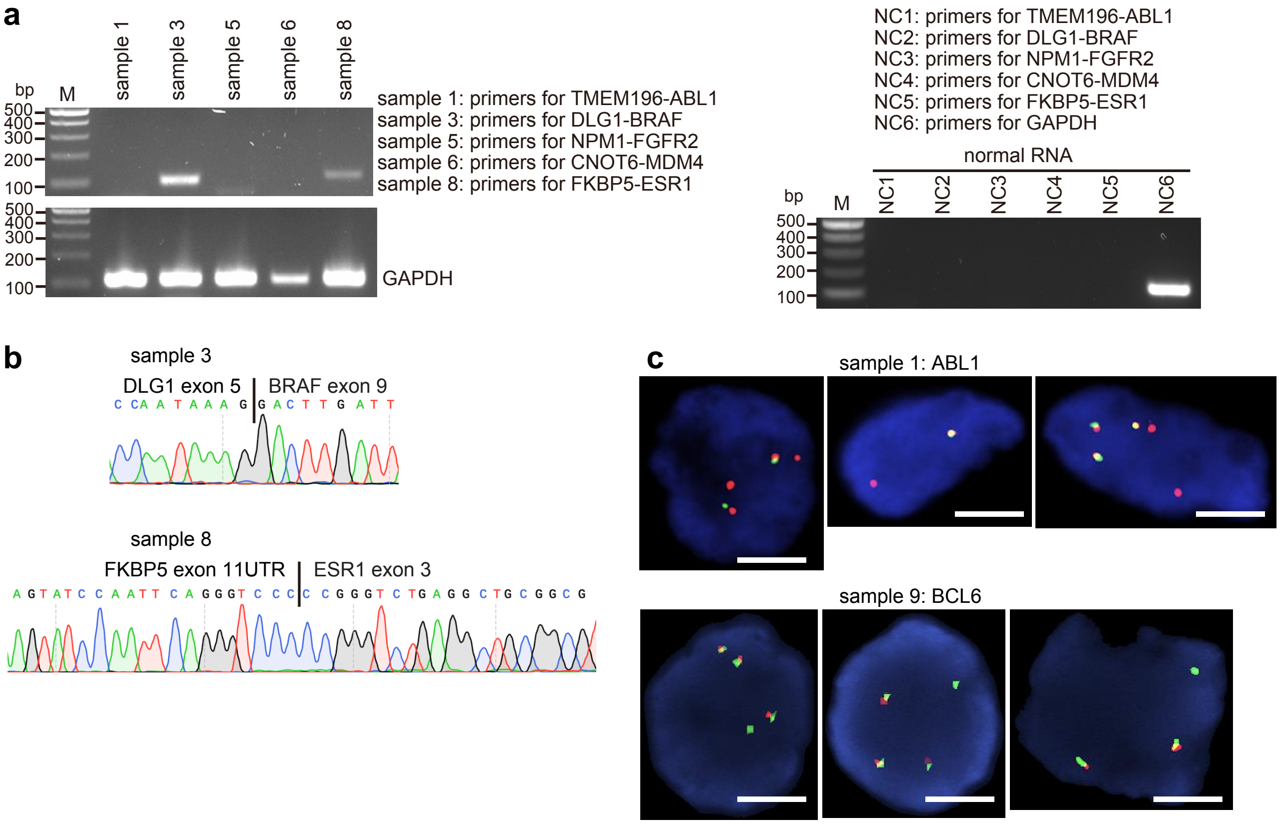

Supplementary Fig.4. Validation of putative fusions by RT-PCR and FISH

(**a**) Images of PCR amplicons of cDNA generated from clinical samples and human normal RNA using indicated primers. M: 100bp-marker. NC: negative control. Strong DLG1-BRAF and faint FKBP5-ESR1 bands were detected. (**b**) Sequencing chromatograms of DLG1-BRAF and FKBP5-ESR1 cDNA. The fusion breakpoint was located in the FKBP5 exon untranslated region (UTR) after stop codon, resulting in production of only FKBP5 protein but not fusion protein. (**c**) Break-apart fluorescence in situ hybridization targeting ABL1 (green: 5' centromeric and red: 3' telomeric) and BCL6 (red: 5' centromeric and green: 3' telomeric). Split signals and loss of either signal indicate the presence of structural variants. White scale bars: 5 µm. Source data of Sup. Fig. 4a are provided as a Source Data file.

Supplementary Fig. 5

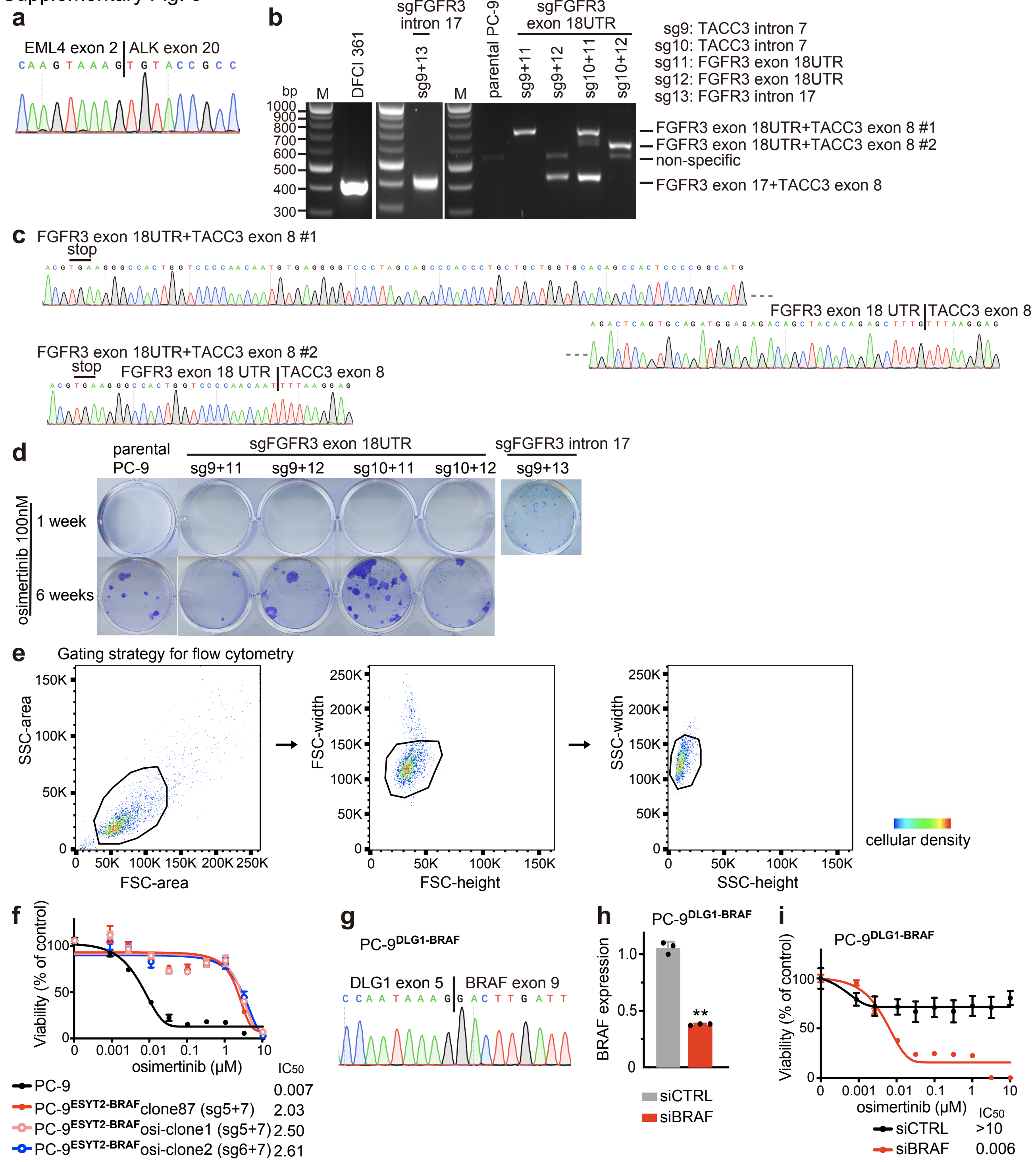

Supplementary Fig. 5. CRISPR-modified PC-9 cells expressing fusion oncogenes

(**a**) Sequencing chromatograms of *EML4-ALK* fusion cDNA derived from bulk CRISPR-modified PC-9 cells. (**b**) Images of *FGFR3-TACC3* PCR amplicons of cDNA generated from CRISPR-modified PC-9 cells and from DFCI 361, a patient-derived xenograft model. DFCI 361 and some CRISPR-modified cell models showed alternative splicing, in which exon 18 of *FGFR3* is skipped. M: 100bp-marker. (**c**) Sequencing chromatograms of *FGFR3-TACC3* cDNA, derived from CRISPR-modified cells without alternative splicing, where stop codons are located before fusion breakpoints. (**d**) Results of colony formation assays following 1 or 6 weeks of treatment with 100 nM osimertinib, using the parental PC-9 cell line or CRISPR-modified PC-9 cells that express *FGFR3-TACC3*. (**e**) Gating strategy used for flow cytometry studies. Pseudo-color represents cellular density. (**f**) Results of cell viability assay after 72 hours of osimertinib treatment. The half maximal inhibitory concentrations (IC50s) are shown for the parental PC-9 cells and for single clones from CRISPR-modified PC-9$^{ESYT2\text{-}BRAF}$ models, selected with or without exposure to 100 nM osimertinib for 1 week (n = 3 biological replicates, mean ± s.d.). (**g**) Sequencing chromatogram of fusion cDNA derived from bulk CRISPR-modified PC-9 cells. (**h**) *BRAF* expression was evaluated by qPCR after treatment with siRNA for 48 hours (n = 3 biological replicates, mean ± s.d., two-sided t test, **p<0.01). (**i**) Cell viability assay after knockdown of *BRAF* by siRNA and 72 hours of treatment with osimertinib (n = 3 biological replicates, mean ± s.d.). Source data of Sup. Fig. 5b, f, h, and i are provided as a Source Data file.

Supplementary Fig. 6

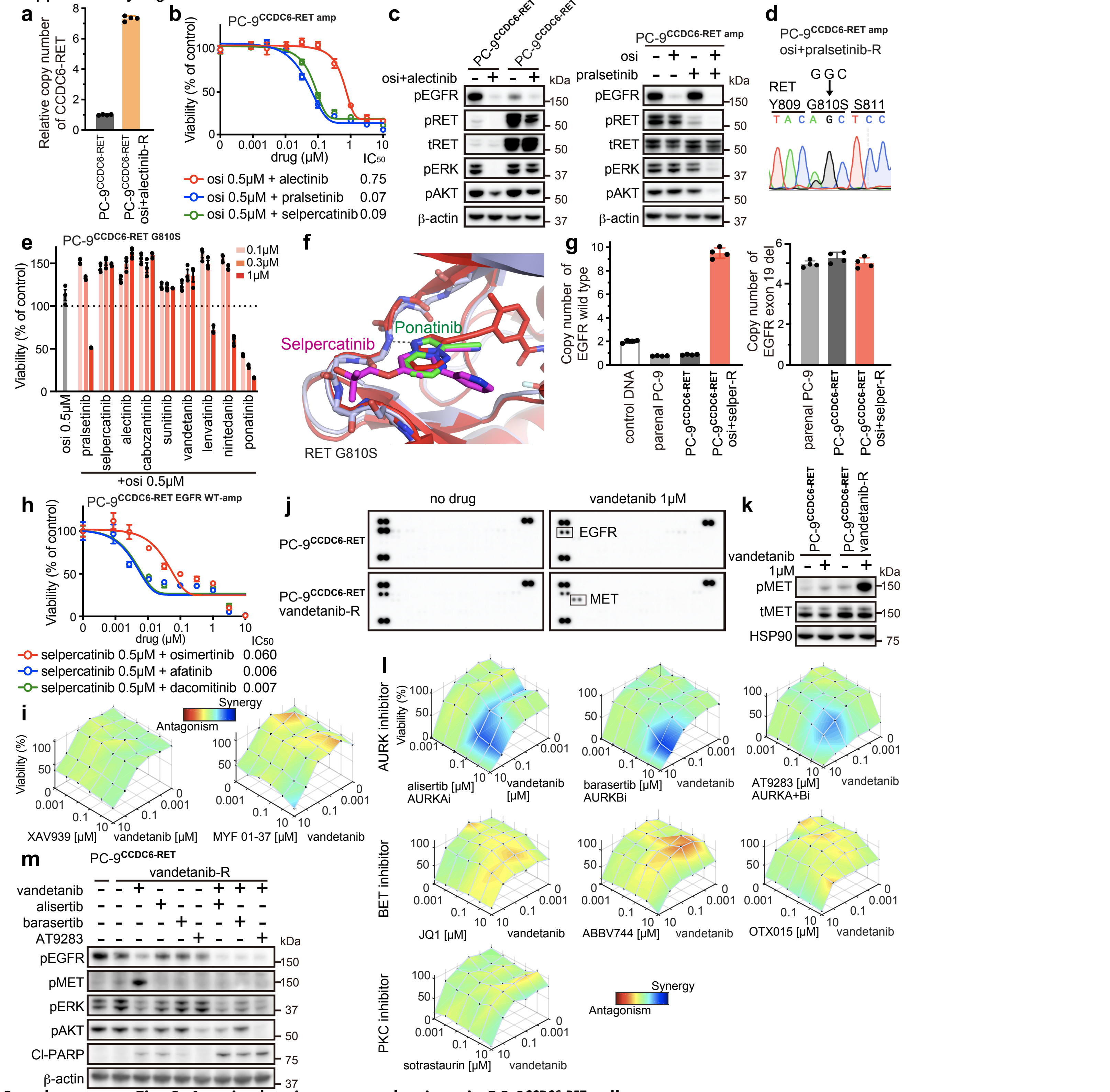

Supplementary Fig. 6. Acquired resistance mechanisms in PC-9$^{CCDC6\text{-}RET}$ cells

(**a**) The relative copy number of *CCDC6-RET* evaluated by qPCR, using *RNaseP* as an internal control (n = 4 biological replicates, mean ± s.d.). (**b**) Cell viability assay after 72 hours of treatment with indicated drugs in PC-9 cell models that harbor *CCDC6-RET* amplification (n = 3 biological replicates, mean ± s.d.). (**c**) Western blot analyses following 48 hours of treatment with 0.5 μM of indicated drugs. (**d**) Sequencing chromatograms of *RET* G810S derived from cDNA *CCDC6-RET* amplicon in the pralsetinib-resistant model. (**e**) Drug screening with use of osimertinib against the *RET* G810S mutation (n = 3 biological replicates, mean ± s.d.). (**f**) Computer-aided docking poses of the truncated analogues of selpercatinib and ponatinib. G810S occurs on the RET hinge region, near the inhibitor binding site. Docking of this hinge-binding fragment into the RET structure shows identical binding modes, as observed in the co-crystal structure of ponatinib bound to KIT (PDB ID 4U01). (**g**) Copy number of wild type *EGFR* and *EGFR* exon 19 deletion, evaluated by qPCR (n = 4 biological replicates, mean ± s.d.). (**h**) Results of cell viability assay after 72 hours of treatment with indicated drugs (n = 3 biological replicates, mean ± s.d.). (**i**) Synergistic inhibitory effects of vandetanib and each drug in the PC-9 cell model with *YAP1* amplification (n = 2 biological replicates, mean). Pseudo-color represents synergy effects. (**j**) Phospho-receptor tyrosine kinase array, following 48 hours of treatment. (**k**) Western blot analyses following 48 hours of treatment. (**l**) Synergistic inhibitory effects of vandetanib and each indicated drug, in vandetanib-resistant PC-9$^{CCDC6\text{-}RET}$ cells (n = 2 biological replicates, mean). Pseudo-color represents synergy effects. (**m**) Western blot analyses following 48 hours of treatment with 1 μM of indicated drugs. Source data of Sup. Fig. 6a-c, e, g-i, and k-m are provided as a Source Data file.

Supplementary Fig. 7

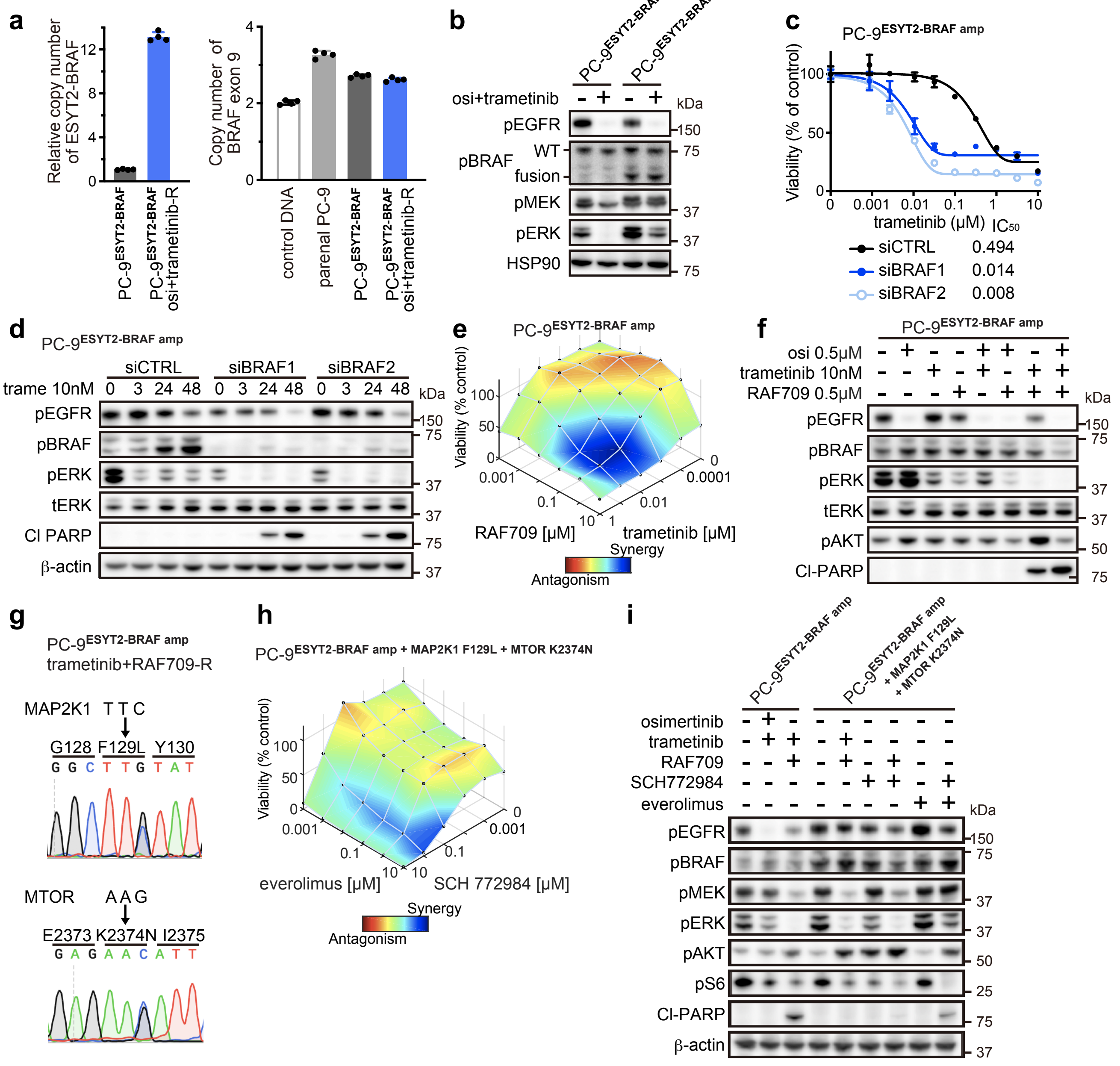

Supplementary Fig.7. Acquired resistance mechanisms in PC-9ESYT2-BRAF cells

(**a**) Relative copy number of *ESYT2-BRAF* and exact copy number of *BRAF* exon 9, evaluated by qPCR using *RNaseP* as an internal control (n = 4 biological replicates, mean ± s.d.). (**b**) Western blot analyses following 48 hours of treatment with 10 nM trametinib and 0.5 µM osimertinib. (**c**) Results of cell viability assays after 72 hours of treatment with the indicated drugs in PC-9 cell models with *ESYT2-BRAF* amplification (n = 3 biological replicates, mean ± s.d.). (**d**) Western blot analyses of PC-9ESYT2-BRAF amp cells treated with 10 nM trametinib over time, and with siRNA for 48 hours. (**e**) Synergistic inhibitory effects of RAF709 and trametinib in PC-9ESYT2-BRAF amp cells (n = 2 biological replicates, mean). Pseudo-color represents synergy effects. (**f**) Western blot analyses after 48 hours of treatment with indicated drugs. (**g**) Representative sequencing chromatograms of *MAP2K1* F129L and *MTOR* K2374N in the DNA of PC-9ESYT2-BRAF amp cells that had acquired resistance to RAF709 plus trametinib. (**h**) Synergistic inhibitory effects in PC-9ESYT2-BRAF amp MAP2K1 F129L + MTOR K2374N cells (n = 2 biological replicates, mean). Pseudo-color represents synergy effects. (**i**) Western blot analyses after 48 hours of treatment with 0.5 µM osimertinib or RAF709, 10a nM trametinib, 1 µM SCH772984, or 50 nM everolimus. Source data of Sup. Fig. 7a-f, h, and i are provided as a Source Data file.

Supplementary Fig. 8

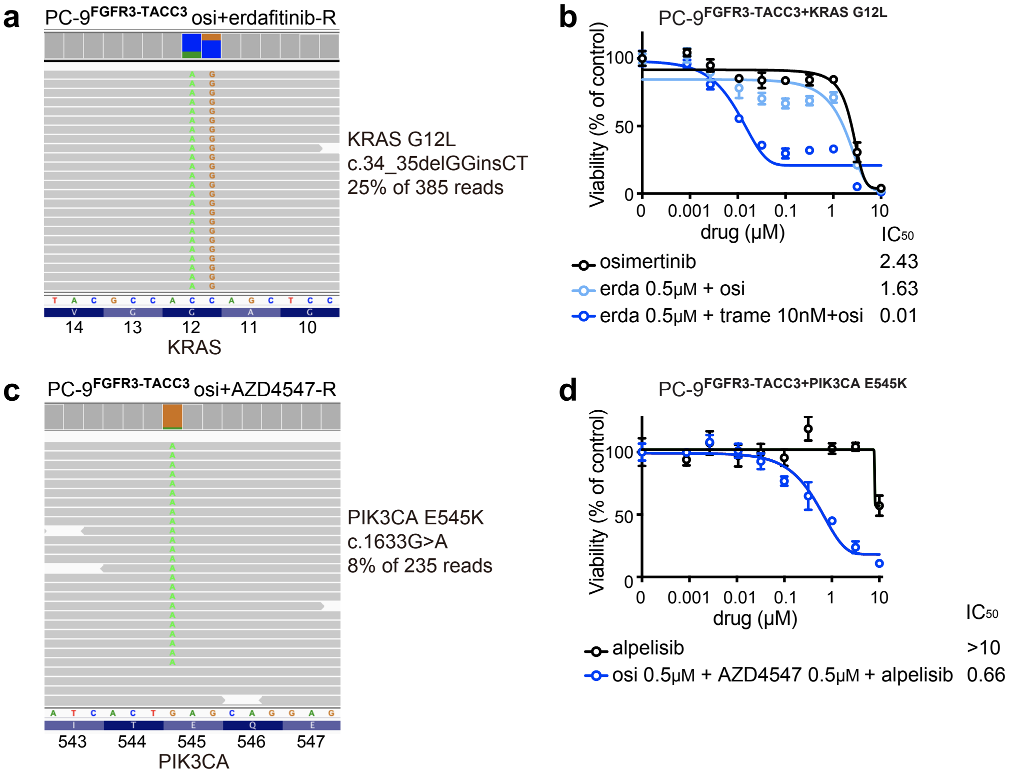

Supplementary Fig.8. Acquired resistance mechanisms in PC-9$^{FGFR3\text{-}TACC3}$ cells

(**a**) *KRAS* mutation detected by DNA-based next generation sequencing OncoPanel in PC-9$^{FGFR3\text{-}TACC3}$ cells that had acquired resistance to osimertinib plus erdafitinib. (**b**) Results of cell viability assays after 72 hours of treatment (n = 3 biological replicates, mean ± s.d.). (**c**) *PIK3CA* mutation detected by OncoPanel, after exposure to osimertinib and AZD4547. (**d**) Results of cell viability assay, following 72 hours of treatment (n = 3 biological replicates, mean ± s.d.). Source data of Sup. Fig. 8b and d are provided as a Source Data file.

Supplementary Table 1

Details of putative fusions detected by DNA-based NGS OncoPanel

SN	putative fusion detected by DNA-based NGS	sequencing depth (AF)	coverage	purity of cancer cell (%)	fusion break points	kinase domain intact	in-frame strand orientation	contig sequence
1-1	EML4-ALK	0.095	201	70	intron 19 of ALK (2:29446862) and intron 2 of EML4 (2:42477047)	yes	in frame	TGAACCAGCAGACTGTGTTGCAAGTATAACCCCACGTGAACGAGGGAGGGAGGGAAGGTTGGGTGGAAGCACCCTGGGTGCCATGGAGCCTAAGCA<br>ACTGGTGCATATATTATGAACACATGCCGTCAAAGGAAAGAATTCTCTGACAGAGGAGAAGGTATATGAAAAATTTA
1-2	LPIN1-ALK	0.164	238	70	LPIN1 (2:11946220) and ALK (2:29446947).	yes	in-frame	TGAGCAAACAAGGTTGGGAACCATGGGCCTGACACTTCCGTCTTGACACTATCTTGACACTCACTAAGTGTGAATGTAACTTGCTCAGTATTTCTGCTG<br>TGCTCAGCCATTGGGTAGGGCAGCTTCAGTGCAATCACAGCAGTGGATTTGAGGGTGCAGCTGGGATCTTGGTCAGTTGTGTT
1-3	DIS3L2-SF3B1	0.176	119	70	DIS3L2 (2:233191624) and SF3B1 (2:198274499)	n/a	no (+/- --> +/+)	CATAGCCTCACCAGGCTTCACGTTCAAGGTCACCAAGAGTGCACTTGTTCACTGTCGAGGGCAGAGGTGACTCCTGGGACTGTGCTCCTGTTTTGGG<br>GGTTTCATCCCATCTGTTTTTACGAGCACTGGAAGTTGCGCCTCCATGGCCTGGTGTCGCATGGCCTGGTGTATCACCTCG
2	TBC1D25-ARAF	0.474	1134	50	TBC1D25 intron 3 and ARAF exon 9 (breakpoints: X:48412000,X:47426385)	yes	in frame	GACCTTCTTACCATTGTACTCAGCCGATTTTTGTCTCTGGAAGCTGCAAGAGTTTTTCTTGCCGGTAGTAGAGGAGGTAGTGATGGAACCCCCCGGGG<br>GAGCCCCAGCCCAGCCAGCGTGTCCTCGGGGAGGAAGTCCCCACATTCAAGTCACCA
3	DLG1-BRAF	0.129	450	80	BRAF intron 8 and DLG1 intron 5 (7:140491094, 3:196900655)	yes	in frame	AAGCTTCTCAAAATTAACACTTAATCCTACTTCCTCACCTGCTGCCTTCTCCTCTTACTGGCCCTCTAAAACAGCTCTCAATGAAGTAAAGATGACGGCAT<br>GGCTGTGTTCCAATAAAACTTTATTTACAAGCCAGGCATGGTGGCTCATGCCTGTAATAATCCCAACACTT
4	ESYT2-BRAF	0.119	218	80	intron 10 of BRAF and intron 8 of ESYT2 (breakpoints 7:140481539, 7:158559016)	yes	in frame	TGCCCATCAGGAATCTCCCAATCATCACTCGAGTCCCGTCTACCAAGTGTTTTCTTGATAAAAACAGTAAAAAAGTCAAGTCAAGCCAAACAGAAAAAGCC<br>GGCTGTTAAGCCCATGCAGCTGAGGGCAGCCAGATCCAGGCTCAGGTGCTCACCTATACTCAGAGCATAAGAGGC
5	TRIM33-ERBB4	nd	nd	40	TRIM33 intron 3 and intron27 ERBB4 (breakpoints: 1:115043594, 2:212252547)	yes	no (-/- --> -/+)	GTGATCCGCCCGCCTCGGCCTCCCAAAGTGCTGGGATTACAGGCGTGAGCCACCGCGCCCGGCCCCAAATTCTTAACTCACTGTTGGCAAAGGCAAA<br>ATGAGGAAATTATGCAGAGACGAGAGAGGAAACATGGTAAGCAAAGACCGAAAATCCTAAA
6	NPM1-FGFR2	0.065	276	20	intron 9 of FGFR2 and exon 1 of NPM1 (10:123263530, 5:170814856)	no	in frame	CTAATTTGGTTTCTGACTTCTGCCAGCCCCCAACCCATTCCTTTCTAAGATTCGATACTCTGCCCTGGTGTGATTCCGTCCTGCGCGGTTGTTCTCTGG<br>AGCAGCGTTCTTTTATCTCCGTCCGCCTTCTCTCCTACCTAAGTGCGTGCCGCCAC
7	CCDC6-RET	0.174	316	30	intron 1 of CCDC6 and intron 11 of RET (breakpoints: 10:61623484, 10:43611062)	yes	in frame	TGTCATATATTTAAGTCTGTATCTCCTTGCTTTCTAAGGTCATTTAACATTTTTATATTTCATTGATATTTCTGATATATGAAAAGCGTGAGGCTGCCCAGA<br>AAGGCCGCACTAGTCTGGGCTGCTGCTGGCAGAGACCACCACCCTAACCCCAGTCAGCTCCAGAGTCACACTCATCAGCACCAGGTCTTGGACCCAT
8	CCDC6-RET	0.073	330	40	intron 11 of RET (10:43611211) and intron 1 of CCDC6 (10:61661624)	yes	in frame	TCCACCACCCTAACCCCAGTCAGCTCCAGAGTCACACTCATCAGCACCAGGTCTTGGACCCATGACTCAACCTCAGTATTTGAGAGGATCAGGTTGATG<br>TATGAGTGAGAATGTGATTGTGGATTATATGATTCAGTGTAGTGAGGGTTATTGTGGGGTGGTGGTCTTAGA
9	CCDC6-RET	0.040	173	50	CCDC6 intron 1 (chr10:61660746) and RET intron 11 (chr10:43610359)	yes	in frame	TTTTGTGTGTGTGTGACAGCTTTATTACCCAGACTGGAGTGCAGTGGTGCGATCTCAGTTCACTGCAACCTCTGGGGACTATGATGAGGTGCCGTTCC<br>CATCTAGGTGAGAGGCAGTGGTCAGGGTCACAGCATCGGGCAGGGGAGCAGC
10	FGFR3-TACC3	0.222	162	50	FGFR3 exon 18 UTR and TACC3 intron 7 (4:1809119, 4:1737404)	yes	in frame	CTAGCAGCCCACCCTGCTGCTGGTGCACAGCCACTCCCCGGCATGAGACTCAGTGCAGATGGAGAGACAGCTACACAGAGCTTTGGTCTGTGTGTGT<br>TAGGGGATGGCAGTCAGACCTGATCACTTGCCCTCTTGTCCCCAGTTTAAGGAGTCGGCCTTGAGGAA
11	FGFR3-TACC3	0.050	260	20	FGFR3 intron 17 (chr4:1808830) :: TACC3 intron 7 (chr4:1737269)	yes	in frame	CCAGAGTGCTGAGGTGTGGGGCGGGCCTTCTGGGGCACAGCCTGGGCACAGAGGTGGCTGTGCGAAGAGGGGCTCGGTGGCACAGCGCTCACCC<br>CGCCAGGATGAAACTCTGACACGCTGTGCTGCTGGGAATGGTGGTGTCTCGGGCAGGGTTGTGGGTGACCGGGGGTGGGA
12	intergenic-ERBB3	0.280	50	20	(6:69262353) and ERBB3 intron 4 (12:56481241)	yes	in frame	AAATGACCATTATCACTGGTCATTAGAGAAATGCAAATCAAAACCACAATGAGATACCATCTCACACTGGTTAGAATGAGCCCTGGCTTTTTGCTTCCCG<br>GGATTGAGGTGCCTGTGTACTGACATCATACCCCGTTGATTAAAACAAGCCTTTCTTAGCCCTGATGGCCCCT
13	FGFR1-intergenic	0.066	227	30	FGFR1 intron 3 (8:38307562) and (8:32056532)	yes	in frame	AGGCAACAGGCCCACTACCGGGGAGGAGGGGTTAGGGCAATTGGCCCTGCTACTTGGCTGCCACCAGCTGTAAGTCAGAGCAAGTAGTAGACTATGA<br>CCGCTCTGCTTATTTTGAACAATCTTTTATTTTTTTCTGGAGTT
14	RET-intergenic	0.088	239	70	RET intron 12 (breakpoints: 10:43612853;10:2360767)	no	no (+/- --> +/+)	CACAGGGTGCTTCCAGATGCCTACCCAGCCCCAGACTGGGTTCACCTCCAAGCCAGTAAAACCCCGGACTCTCCAGGGGCACAGGTAGAGTATATAGT<br>ATATATACTGGCCTACACCTTGATATGGGACTTCCAGCCTCCAGGACGGTGAGGAAACA
15	ROS1-intergenic	0.062	65	20	non-gene region and intron32 of ROS1 (breakpoints 6:82962600 and 6:117647860)	no	in frame	CACAAAAAAAAAAAAAGAAAAGAAAACATACTTAACATACTTAAACTTTCTCCAGAATTCCAAACCTATAAGACAATTATTCCATGATTTTTAACTGGGA
16	TMEM196-ABL1	0.101	388	40	TMEM196 intron 1 and ABL1 intron 1 (breakpoints: 7:19784826; 9:133654216)	yes	in frame	CAATTCAACCAACTGCAGATTTTTTAAAAATTGAGAAAAATGATAAAAATAAGAGTACAACAATAAAAATAATACACATTGTTATACTATATTGCATCATTTCA<br>ATTCCTGTGATATTATGGATTCCTACGGGTAGGAAAAACACAGCCCATTCAACCAAATATACTTTAACCCCTAGAGATTTAGCT
17	CUL4B-BCORL1	0.325	265	40	CUL4B intron 2 and BCORL exon 6 (X:119703172, X:129159099)	n/a	no (-/+ --> +/+)	GCCGGCAGACCCATCATGTTCTACTCCCTTAGTATACAAAGACAACCCTTGCCCTCCATTCTCAATTGGCAATGTAGGTTTAGAGCTATCCCAGTATCGC<br>AGCCACCATGCCCAGGACAAGTCTCTGCTGAGCCAGGGCCGAAGGCACCTGTGGCGAGCCCGAGAAATGCCCTGGAGGACAGAGGCT
18	BCORL1-DIAPH2	0.068	221	20	BCORL1 intron 1 and DIAPH intron 17 (X:96300472, X:129146489)	n/a	in frame	AGTTATAGCTAGAGGTATCAATTTGAGATCATTATCACATCGATGGTATTCACATAATACATCGGGTTGAACCCAGAAGGTGGTGTTGGCTCAAATCCTG<br>GGCCTCATGTCCCTTCTCTGGTTGTATCTTCCATGCTAGAAGAGCACCTCTTTCTGATGAGGAGTCAACGACA
19	IGH-CCND1	0.125	32	30	placing the CCND1 gene adjacent to the IgH enhancer region (11:69468179, 14:106330254)	n/a	no (+/- --> +/+)	TGTTTTACAATGTCATATACTGCCATGTACTAGTTTTAGTTTTCTCTTAGAACATTGTATTACAGATGCCTTGGAGCCCTTGCGCCTCGTCTGTGTGGCC<br>GCTGTTGCCTCAGGGCATCCTCCTGAGCCCCCCAGGCTGCTCCGGGGCT
20	TDG-CTNND2	0.151	245	50	TDG intron 5 and CTNND2 intron 2 (12:104376730, 5:11580497)	n/a	in frame	ACCAGGGAAGTATGGTATTGGATTTACCAACATGGTGGAAAGGACCACGCCCGGCAGCAAAGATCTCTCCAGGTAAGTACACAGCATTTGATAATAAGA<br>AAGTTAGATTATTCCCATAAATATGTATTCATTCTTTACATAAAACTCCATGGAAAATACCTTA
21	FKBP5-ESR1	0.049	182	40	FKBP5 exon 12 UTR and ESR1 exon 3 (breakpoints 6:35543300, 6:152129289)	n/a	in frame	TTAAGTCCAGCTCATTTCAGTTTCTATCAACCTTCAAGTATCCAATTCAGGGTCCCCCGGGTCTGAGGCTGCGGCGTTCGGCTCCAACGGCCTGGGG<br>GGTTTCCCCCCACTCAACAGCGTGTCTCCGAGCCCGCTGATGCTACT
22	SLC4A4-GNAS	0.046	197	20	intron 3 of SLC4A4 and exon 1 of GNAS (breakpoint 4:72167270 and 20:57428283).	n/a	no (+/+ --> -/+)	TTGCAGTCACTATGCAAGAGCCCGAATAGCTCTAGATGCTATGAGGGAGGCAGAATAAGGAGCTGGGGCCTGCAGGTAGAGAGCCCCAAACTTATTTT<br>GAGAGGCCGCCACCGTGTTATGGGCGTGCGCAACTGCCTCTACGGCAATAATATGTCAGGACAACGCGATATCCCC
23	GNAS-SYNDIG1	0.021	1530	50	GNAS exon 1 and SYNDIG1 intron 1 (breakpoints: 20:57428552, 20:24503480)	n/a	no (+/+ --> +/-)	TGGAGACCGAACCGCCTCACAACGAGCCCATCCCCGTCGAGAATGATGGCGAGGCCTGTGGACCCCCAGAGGTCTCCAGACCCAACTACCAGGTGAC<br>ATGTTGGCTGCCACTTCCCCTTCTGCCATGATTAAAAGCTTCCTGAAGCTCCCCCAGAAGCCAAGTAGATGCTG
24	JAK2-BNC2	0.563	16	75	intron 19 of JAK2 and intron 3 of BNC2 (breakpoints: 9:5089315, 9:16708809)	no	no (+/- --> +/+)	AGAGTTCCTTAATATAAGCTTAAAGATAGTCTGCTGCTGGGCATGGTGGCTCATGCCTGTAATCCCAGCACTTTGGGAGGCCGAGTTGGGTGGCAGG<br>CAGCTGGCCTGAGATGCTGATTCAGCGCTTTAACAA
25	CNOT6-MDM4	0.085	719	30	CNOT6 intron 13 and MDM4 exon 4 (breakpoint: 5:180000930, 1:204499843)	n/a	in frame	TAGTACAGACCTCATCCTACAAGCTAGTAAGTTTCACTTGTTCTGTTCTGTTCACCAAACATGACATAAAGGCATGTCTTGGAAGCAACTTTATGATCAGC<br>AGGAGCAGCATATGGTATATTGTGGTGGAGATCTTTTGGGAGAACTACTGGGACGTCAGAGCTTCTCCGTGAAAGAC
26	MYC-FBXO32	0.094	413	30	MYC exon 3 and FBXO32 intron 4 (breakpoints 8:128752768, 8:124537716)	n/a	in frame	GTTGTTTCTGTGGAAAAGAGGCAGGCTCCTGGCAAAAGGTCAGAGTCTGGATCACCTTCTGCTGGAGGCCACAGCAAACCTCCTCACAGCCCACTCTC<br>CTAGCTCTCTCTGTAGAATGAGAATAAGCCCAAGTCTACAGTAACTGTAGCTACCATTAACAGCCCAGAAAGGT
27	NRG1-EBF2	0.245	163	60	NRG1 intron 5 and EBF2 (8:32536874; 8:25864028)	n/a	(+/- --> +/+)	TTCCAGAGCATAATGCCAGCGATTTAACTGTATGGATAGTAGTTAAATCCTTAACAATATATAGTACATGTTCATTTTCTAAATAGTAGGGTTGTAATTTTC<br>TGTGGCATTATTCCTTATGACTCTATATTTAAATAAACAACCTTCTATATTCTTCCTGATGAGAAAACTA
28	WBP1L-NT5C2	0.102	372	50	WBP1L intron 1 and NT5C2 exon 18 (breakpoints: 10:104551774, 10:104849287)	n/a	in frame	TGGAAAGGATAAACAGTATGCCTTTTGCACATATGCAAAAAATTATCTCCTCTCTTTGGTGGCAGTATAAACTGTGGGACAAAAATCTTCAGAAACTTTTC<br>AGACGTACCTTTCATGGAGCCCCCTCCCTCCCCCGAGTAGAACCCTAACAGGGACCTCGTTTGTTCCTGTGAG
29	RARA-TADA2A	0.058	226	30	RARA intron 2 and TADA2A intron 11 (breakpoints: 17:38498242, 17:35826819)	n/a	no (+/+ --> +/-)	TACTCTGTGTACTCCTCATCTGGAGCCTTTCCCCCTTCCTGCTTCTCTCCTCTCCTCCCCCTTCCCAGGCTGCCCCCAGGTGTGTCCCAAACTGGGAC<br>AGGAAGTAAAGGATCAGTCACCTGGTACAAACCACACAAAGAATGCT
30	BCL6-intergenic	0.026	453	25	BCL6 intron 1 (breakpoints: 3:187454141, 17:22112634)	n/a	no (-/+ --> +/+)	GACAAAAGAACCTATCTCCTCCTCTCCCCAGCCCAACTCGAGATAGGCAAAGAGAGATAGACTAACTCGGTCTTTACCAAACTTCAAGGATCATGAATAA<br>CTTAGAAATGTGATGCTTAATTTCCAAACATTTGTTAACTTTCA
31	MAP3K1-intergenic	0.057	953	50	MAP3K1 exon 5 (breakpoints: 5:56161211, 12:65338814)	no	no (+/- --> +/+)	CTTGTGAACCACAGATGTATTAACCTATAGGTTTTCTTTTTAACTCTTTAGAACTGCAGCTGTGCACGTGGAACATTCTGTATTCATCTGCTATTTTAGTG<br>GTTGCCTGAAACATCCCGAGAGGAAGTCAGGGAGAGAGAATACCATCTGCTAGGAGTGAGCAGAGTTCACATGAGT
32	MTOR-intergenic	0.066	211	50	MTOR intron 32 (breakpoints: 1:11205147, 1:14402128)	yes	no (-/+ --> +/+)	CTATGTGCAGAATAGTTGACACTTACCCCATATGCCCGACTGTAACTCTCTCCTGCCATTGCAGTTAATTCAGCATCCAGCAGGTCCCTGGCCTTGTCA<br>ATGCACTAGAAGAGAAACAACCCTTGGGACTGAGCTCTGGACTTGGGAGCAAAAACGCAGTTCCTTGAAAAATGATTGTTTTTGCTCTTTCCCGGGGAA
33	NOTCH2-intergenic	0.136	59	30	NOTCH2 intron 8 (breakpoints: 1:18210823, 1:120509822)	n/a	in frame	TGCAAACTTCCCCCCACCCCACCCCGCCACACATAAGAGCTGGTGACATAACAGGCTTGTTTAATTTGGTCTACATAGCTTGATGAATTCTAGCACTGCC<br>TTACTCTTTGAGTAAGGAAGGTTTGCTTGTGAACCCTGAC
34	RARA-intergenic	0.087	287	50	RARA intron 2 (breakpoints: 1:34687096, 17:38497840)	n/a	in frame	CAAAGTGAAAGAAGAAGATGTACATTGAAGACTATCCCTATGAAATTTCAGAAGAGCAAGGAGGCGAGCGGGTGATGTCACGGGCAGCGGTGGGTGG<br>GTCACTCGGAGGTGAGGCGCCGCCAGGCGAGTTCAGCGAGAGTTCAGCCGCATTGCAT
35	RARA-intergenic	0.145	338	60	RARA intron 2 (breakpoints: 17:38496646, 17:41185541)	n/a	in frame	TTTTTACACTGTAAATCTGATCTTTCCTTTGCTTGGAACCTTTCTATGGCTTTTCCTTGCATTTAGGATAAAATCCAAAAGCCTTACTGAGGCCCCTTGTG<br>ACTCTGGCCCTCCCAGGGGGTAGTCGTGGGAAGAGCTTCAGGTTCAGAGGCAGAAGTCCCAGG

Supplementary Table 2
Quality check metrics of RNA-seq

sample ID	putative fusion detected by DNA-based NGS	Reads Aligned in Pairs	mRNA Bases % (RNA)	Product	Coding Bases % (RNA)	Coding Bases (RNA)	Contamination %	Correct Strand Reads % (RNA)	Correct Strand Reads (RNA)	Duplication % (Library AVG)	Duplication % (Library MAX)	Duplication % (Library MIN)	Incorrect Strand Reads (RNA)	Intergenic Bases % (RNA)	Intergenic Bases (RNA)	Intronic Bases % (RNA)	Intronic Bases (RNA)	Mean Read Length	Median 3Prime Bias (RNA)	Median 5Prime Bias (RNA)	Median 5Prime-3Prime Bias (RNA)	Median CV Coverage (RNA)
sample 1	TMEM196-ABL1	61,272,682	90.8	Transcriptome Capture v1	81.5	3,792,782,376	4.37	98.9	53,533,404	37.48	37.48	37.48	613,372	4	178,816,972	5	233,648,980	76	0	0.02	0	1.1
sample 2-1	FGFR1-intergenic	181,728,542	86.5	Transcriptome Capture v1	74.3	10,267,044,236	3.75	99.2	150,863,920	61.68	61.68	61.68	1,174,683	7	972,963,207	6	810,807,209	76	0	0.09	0.18	0.8
sample 2-1	FGFR1-intergenic	109,669,086	88	Transcriptome Capture v1	76	6,353,423,390	4.63	99	92,419,924	63.187	63.187	63.187	973,147	6	520,310,360	6	461,291,593	76	0	0.04	0	0.9
sample 3	DLG1-BRAF	61,006,724	88.7	Transcriptome Capture v1	77.6	3,604,245,309	5.01	98.8	51,833,428	82.484	82.484	82.484	618,491	5	246,563,914	6	264,930,790	76	0	0.05	0	0.9
sample 4	EBF2-NRG1	14,337,242	90.7	Transcriptome Capture v1	79.3	863,109,993	6.8	98.7	12,521,826	64.347	85.662	43.031	168,267	4	48,008,926	5	51,240,181	76	0	0.02	0	0.9
sample 5	NPM1-FGFR2	149,863,560	88.9	Transcriptome Capture v1	79.4	9,167,346,139	6.07	98.9	130,304,930	62.499	62.499	62.499	1,439,478	6	662,174,594	5	583,585,048	76	0	0.04	0.04	0.9
sample 6	CNOT6-MDM4	158,861,854	75.4	Tru-Seq Strand Specific RNA Sequencing v1	47.2	7,559,166,741	1.62	99.7	114,195,080	63.59	63.59	63.59	394,652	14	2,191,038,537	8	1,329,929,647	101	0.18	0.25	1.49	0.5
sample 7	SLC4A4-GNAS	69,716,012	88.7	Transcriptome Capture v1	75.4	3,980,646,665	2.87	98.4	59,276,060	48.698	48.698	48.698	987,016	5	271,167,927	6	313,357,588	76	0	0.03	0	1.1
sample 8	FKBP5-ESR1	92,034,156	89.8	Transcriptome Capture v1	78.9	5,573,904,845	3.17	98.4	79,871,986	62.938	62.938	62.938	1,260,208	5	378,755,348	5	321,054,260	76	0	0.03	0	0.9
sample 9	BCL6-intergenic	44,430,942	84.2	Transcriptome Capture v1	72.9	2,467,486,379	2.68	98.4	36,339,120	74.367	74.367	74.367	604,869	9	310,261,877	6	209,677,600	76	0	0.04	0	1
sample 10	RET-intergenic	88,843,590	79.1	Transcriptome Capture v1	66.6	4,498,145,926	3.47	98.1	67,049,630	98.374	98.374	98.374	1,264,869	11	752,205,645	7	461,753,836	76	0	0	0	1.3
sample 11	SYNDIG1-GNAS	8,818,222	69.2	Transcriptome Capture v1	62.8	445,976,565	0.51	98.5	6,213,766	98.086	98.086	98.086	93,189	18	129,545,829	12	84,751,890	76	0	0	0	2.9

sample ID	putative fusion detected by DNA-based NGS	PF Bases (BC)	PF Bases (RNA)	PF Bases Aligned	PF Bases Aligned (RNA)	PF HQ Aligned Bases	PF HQ Aligned Q20 Bases	PF HQ Aligned Reads	PF Mismatch Rate	PF Noise Reads	PF Reads	PF Reads %	PF Reads Aligned	PF Reads Aligned %	PF Reads Aligned (Paired)	Reads Aligned in Pairs %	Ribosomal Bases % (RNA)	Ribosomal Bases (RNA)	Total Reads	UTR Bases % (RNA)	UTR Bases (RNA)	Usable Bases % (RNA)
sample 1	TMEM196-ABL1	4,759,086,104	4,759,086,104	4,652,371,771	4,652,371,771	4,301,823,107	4,242,165,270	56,930,723	0	216	62,619,554	97.7	61,658,737	98.5	61,658,737	99.4	0.4	17,663,433	64,062,774	9.2	429,460,010	88.7
sample 2-1	FGFR1-intergenic	14,013,558,376	14,013,558,376	13,818,808,091	13,818,808,091	12,529,784,941	12,271,468,405	165,431,946	0	926	184,388,926	96.6	182,571,702	99	182,571,702	99.5	0.6	77,289,416	190,858,632	12.2	1,690,704,023	85.3
sample 2-1	FGFR1-intergenic	8,513,995,456	8,513,995,456	8,359,072,482	8,359,072,482	7,620,165,642	7,477,178,548	100,612,982	0	461	112,026,256	97.8	110,442,846	98.6	110,442,846	99.3	0.2	20,777,968	114,580,846	12	1,003,269,171	86.4
sample 3	DLG1-BRAF	4,969,644,408	4,969,644,408	4,645,872,592	4,645,872,592	3,632,163,940	3,567,436,830	48,684,887	0	501	65,390,058	97	62,952,993	96.3	62,952,993	96.9	0.3	11,961,336	67,433,670	11.2	518,171,243	83
sample 4	EBF2-NRG1	160,968	1,117,099,832	1,088,028,019	1,088,028,019	993,435,559	972,259,788	13,180,307	0	228	14,698,682	95.7	14,473,244	98.5	14,473,244	99.1	0.2	2,193,008	15,366,964	11.3	123,475,911	88.3
sample 5	NPM1-FGFR2	11,999,590,904	11,999,590,904	11,541,362,564	11,541,362,564	10,168,226,774	9,637,073,236	134,659,785	0	1,904	157,889,354	93.8	153,126,097	97	153,126,097	97.9	0.3	29,980,821	168,281,664	9.5	1,098,275,962	85.5
sample 6	CNOT6-MDM4	16,298,474,434	16,298,474,434	16,021,320,322	16,021,320,322	15,196,362,970	14,983,190,343	151,393,594	0	1,253	161,371,034	100	160,008,386	99.2	160,008,386	99.3	2.6	420,329,531	161,371,034	28.2	4,520,855,866	74.1
sample 7	SLC4A4-GNAS	5,420,283,368	5,420,283,368	5,282,044,121	5,282,044,121	4,844,920,217	4,613,500,645	64,244,409	0	1,534	71,319,518	94.2	70,185,215	98.4	70,185,215	99.3	0.2	11,296,564	75,745,272	13.4	705,575,377	86.5
sample 8	FKBP5-ESR1	7,294,448,536	7,294,448,536	7,068,235,020	7,068,235,020	6,404,594,775	6,288,397,560	84,773,163	0	703	95,979,586	96.8	93,664,797	97.6	93,664,797	98.3	0.3	24,285,796	99,161,876	10.9	770,234,771	87
sample 9	BCL6-intergenic	3,517,274,224	3,517,274,224	3,382,744,744	3,382,744,744	2,998,160,170	2,961,717,305	39,753,917	0	1,903	46,279,924	98.6	44,946,998	97.1	44,946,998	98.9	0.4	14,004,578	46,922,478	11.3	381,314,310	81
sample 10	RET-intergenic	6,880,254,008	6,880,254,008	6,749,411,689	6,749,411,689	5,701,168,743	5,611,512,571	75,395,681	0	544	90,529,658	97.3	89,319,609	98.7	89,319,609	99.5	2.9	193,421,523	93,072,714	12.5	843,884,759	77.6
sample 11	SYNDIG1-GNAS	818,202,168	818,202,168	710,689,786	710,689,786	561,829,832	549,481,061	7,558,611	0	1,082,690	10,765,818	97.7	9,608,584	89.3	9,608,584	91.8	0.7	4,794,714	11,016,834	6.4	45,620,788	60.1

Supplementary Table 3

Candidate fusions detected by at least 2 fusion callers based on the RNA-seq data

sample number	fusions detected by DNA-base Oncopanel	putative fusions detected by RNA-seq and at least 2 fusion callers
1	TMEM196-ABL1	ABCA13--ZPBP
		AKAP9--CNTNAP2
		CNTNAP2--AKAP9
		CTSC--RAB38
		HDAC9--CNTNAP2
		KANSL1--ARL17A
		KANSL1--ARL17B
		NCOR1--TPX2
		PON3--AC099342.1
		RP11-89K10.1--ANXA13
		SAMD5--SASH1
		TAF2--TRPS1
		TRIM2--FSTL5
2-1	FGFR1-intergenic	AKR1C1--AKR1E2
		AP3S1--AQPEP
		APLP2--RNASE1
		BNIP3L--DPYSL2
		C10orf112--PLXDC2
		C5orf56--RAD50
		CHD9--SNTB2
		CMTM8--CMTM7
		CTC-786C10.1--RP11-680G1
		CTD-2337A12.1--CAST
		EIF4E3--FOXP1
		HM13--NAV2
		LINC00670--MYOCD
		METTL13--DNM3
		NDUFV3--PKNOX1
		PARVA--VAPB
		PFKFB3--RP11-563J2.2
		PVT1--C8orf47
		RP1-34H18.1--NAV3
		RP11-120D5.1--MID1
		RP11-123O10.4--GRIP1
		RP11-141M1.3--STARD13
		RP11-275N1.1--NEBL
		RP11-381K20.2--KLHL3
		RP11-444D3.1--SOX5
		RP11-680G10.1--GSE1
		RP11-98I9.4--TSTD3
		RP3-323P24.3--FAAH2
		SAMD5--SASH1
		TCF3--AC009120.4
		TMED3--KIAA1024
		TULP4--RP11-732M18.3
2-2	FGFR1-intergenic	C5orf56--RAD50
		HM13--NAV2
		LINC00670--MYOCD
		PVT1--C8orf47
		RP1-34H18.1--NAV3
		RP11-123O10.4--GRIP1
		RP11-275N1.1--NEBL
		RP11-444D3.1--SOX5
		RP11-680G10.1--GSE1
		RP3-323P24.3--FAAH2
		SAMD5--SASH1
		TCF3--AC009120.4
		TULP4--RP11-732M18.3
3	DLG1-BRAF	BRAF--DLG1
		DLG1--BRAF
		PFKFB3--RP11-563J2.2
		RP11-123O10.4--GRIP1
4	EBF2-NRG1	RP11-123O10.4--GRIP1
		WWC1--RARS
5	NPM1-FGFR2	CTSC--RAB38
		EIF4E3--FOXP1
		NDUFV3--PKNOX1
		PFKFB3--RP11-563J2.2
		RP1-34H18.1--NAV3
		RP11-123O10.4--GRIP1
		RP11-444D3.1--SOX5
		RP11-680G10.1--GSE1
		SAMD5--SASH1
		TULP4--RP11-732M18.3
6	CNOT6-MDM4	ATF6--ATP2B4
		CTSC--RAB38
		DLG1--BRAF
		RABGAP1L--FGF18
		RUFY1--LGR6
		YY1AP1--DUSP12
7	SLC4A4-GNAS	AKR1C1--AKR1E2
		C5orf56--RAD50
		MCF2L--DYNC1I1
		RP11-680G10.1--GSE1
		TNS3--SUN3
8	FKBP5-ESR1	AHR--TMEM39A
		KANSL1--ARL17A
		KANSL1--ARL17B
		PFKFB3--RP11-563J2.2
		RP1-34H18.1--NAV3
		RP11-123O10.4--GRIP1
		RP11-444D3.1--SOX5
		RP11-680G10.1--GSE1
9	BCL6-intergenic	C9orf3--SORCS1
		PLEKHA7--TP53I11
10	RET-intergenic	no
11	SYNDIG1-GNAS	no

Supplementary Table 4

Summary of mechanisms of acquired resistance to inhibition of EGFR and fusion genes

fusion	initial drug	resistant mechanism	strategy to overcome
CCDC6-RET	alectinib + osi	amplification of *CCDC6-RET*	selpercatinib or pralsetinib + osi
	pralsetinib + osi	*RET* G810S	ponatinib + osi
	selpercatinib + osi	amplification of wild type *EGFR*	afatinib or dacomitinib + osi
	vandetanib	amplification of *YAP1*	alisertib + vandetanib
ESYT2-BRAF	trametinib + osi	amplification of *ESYT2-BRAF*	RAF709 + trametinib
	RAF709 + trametinib	*MAP2K1* F129L + *MTOR* K2374N	SCH772984 + everolimus
FGFR3-TACC3	erdafitinib + osi	*KRAS* G12L	trametinib + erdafitinib + osi
	AZD4547 + osi	*PIK3CA* E545K	alpelisib + AZD4547 + osi

Supplementary Table 5

Time to acquiring resistance to drugs in PC-9 CCDC6-RET models

cell line	drugs	time to resistance		resistant mechanism
		days	(months)	
PC-9 $^{CCDC6\text{-}RET}$	osimertinib + alectinib	73	(2.4)	amplification of *CCDC6-RET*
PC-9 $^{CCDC6\text{-}RET}$	vandetanib	73	(2.4)	amplification of *YAP1*
PC-9 $^{CCDC6\text{-}RET}$	osimertinib + selpercatinib	122	(4.1)	amplification of wild type *EGFR*
PC-9 $^{CCDC6\text{-}RET}$ osi+alectinib-R	osimertinib + pralsetinib	178*	(5.9)*	*RET* G810S

*only duration of treatment with osi/pralsetinib
