# Supplementary material for: Genomic and biological study of fusion genes as resistance mechanisms to EGFR inhibitors
Source: Nat Commun. 2022 Sep 24;13:5614. doi: 10.1038/s41467-022-33210-2 (PMC9509394; doi:10.1038/s41467-022-33210-2)
Supplement: Supplementary file 3 — Reporting Summary [file 41467_2022_33210_MOESM3_ESM.pdf]

Corresponding author(s): Pasi A. Jänne

Last updated by author(s): Sep 7, 2022

Reporting Summary

Nature Research wishes to improve the reproducibility of the work that we publish. This form provides structure for consistency and transparency in reporting. For further information on Nature Research policies, see our Editorial Policies and the Editorial Policy Checklist.

Statistics

For all statistical analyses, confirm that the following items are present in the figure legend, table legend, main text, or Methods section.

n/a	Confirmed	
☐	☒	The exact sample size (*n*) for each experimental group/condition, given as a discrete number and unit of measurement
☐	☒	A statement on whether measurements were taken from distinct samples or whether the same sample was measured repeatedly
☐	☒	The statistical test(s) used AND whether they are one- or two-sided<br>*Only common tests should be described solely by name; describe more complex techniques in the Methods section.*
☐	☒	A description of all covariates tested
☒	☐	A description of any assumptions or corrections, such as tests of normality and adjustment for multiple comparisons
☐	☒	A full description of the statistical parameters including central tendency (e.g. means) or other basic estimates (e.g. regression coefficient) AND variation (e.g. standard deviation) or associated estimates of uncertainty (e.g. confidence intervals)
☐	☒	For null hypothesis testing, the test statistic (e.g. *F*, *t*, *r*) with confidence intervals, effect sizes, degrees of freedom and *P* value noted<br>*Give P values as exact values whenever suitable.*
☒	☐	For Bayesian analysis, information on the choice of priors and Markov chain Monte Carlo settings
☒	☐	For hierarchical and complex designs, identification of the appropriate level for tests and full reporting of outcomes
☒	☐	Estimates of effect sizes (e.g. Cohen's *d*, Pearson's *r*), indicating how they were calculated

Our web collection on statistics for biologists contains articles on many of the points above.

Software and code

Policy information about availability of computer code

Data collection	EPSON perfection V750 pro (colony formation assay), BIO-RAD UNIVERSAL HOOD II Gel Documentation System with CFW-1312M Camera (images of PCR bands), StepOnePlus Real-Time PCR System (Quantitative RT-PCR data), FLUOstar Omega (Cell growth-inhibition assay), Amersham Imager 600 (Western blot), BD LSR Fortessa flow cytometer (flow cytometry),IncuCyteS3 Live-Cell Imaging Analysis System (Essen Bioscience)
Data analysis	SnapGene 4.1.9 (sequencing chromatogram), StepOne Software v2.3 (Quantitative RT-PCR), ImageQuant TL1D v8.2 (Western blot analysis), GraphPad Prism v9.0.0, SAS 9.4, Integrative Genomics Viewer (IGV) (version 2.8.0), FlowJo v10 (FlowJo, LLC), Combenefit v.2.021

For manuscripts utilizing custom algorithms or software that are central to the research but not yet described in published literature, software must be made available to editors and reviewers. We strongly encourage code deposition in a community repository (e.g. GitHub). See the Nature Research guidelines for submitting code & software for further information.

Data

Policy information about availability of data

All manuscripts must include a data availability statement. This statement should provide the following information, where applicable:

- Accession codes, unique identifiers, or web links for publicly available datasets
- A list of figures that have associated raw data
- A description of any restrictions on data availability

Raw fastq files of RNA-seq data from clinical samples generated in this study have been deposited in the NCBI GEO database under accession code GSE182323 [https://www.ncbi.nlm.nih.gov/geo/query/acc.cgi?acc=GSE182323]. Protein data were obtained from PDB ID 6NEC [https://www.rcsb.org/structure/6NEC] and 4U0I [https://www.rcsb.org/structure/4U0I]. Analyzed OncoPanel data in this manuscript were deposited into AACR GENIE project for public access [https://

www.aacr.org/professionals/research/aacr-project-genie/aacr-project-genie-data/] but access to raw fastq files of OncoPanel is restricted to investigators approved by the DFCI IRB. Source data are provided with this paper.

Field-specific reporting

Please select the one below that is the best fit for your research. If you are not sure, read the appropriate sections before making your selection.

☒ Life sciences ☐ Behavioural & social sciences ☐ Ecological, evolutionary & environmental sciences

For a reference copy of the document with all sections, see nature.com/documents/nr-reporting-summary-flat.pdf

Life sciences study design

All studies must disclose on these points even when the disclosure is negative.

Sample size	A sample size of two to four replicates was selected for in vitro experiments, which were then confirmed via independent replication studies. Quantitative proteomics experiments were performed using n=2 biologically independent samples, which was expected to yield significant results based on previous experience.
Data exclusions	No data were excluded.
Replication	All experiments have been performed in at least two independent experiments. All replication attempts were successful.
Randomization	Wells of cells were randomly assigned to control or treatment groups.
Blinding	RNA sequencing was blinded. All other experiments were not blinded; blinding was not applicable to this study as data collection and analysis was not prone to bias.

Reporting for specific materials, systems and methods

We require information from authors about some types of materials, experimental systems and methods used in many studies. Here, indicate whether each material, system or method listed is relevant to your study. If you are not sure if a list item applies to your research, read the appropriate section before selecting a response.

Materials & experimental systems

n/a	Involved in the study	
☐	☒	Antibodies
☐	☒	Eukaryotic cell lines
☒	☐	Palaeontology and archaeology
☒	☐	Animals and other organisms
☐	☒	Human research participants
☒	☐	Clinical data
☒	☐	Dual use research of concern

Methods

n/a	Involved in the study	
☒	☐	ChIP-seq
☐	☒	Flow cytometry
☒	☐	MRI-based neuroimaging

Antibodies

Antibodies used	All antibodies were described in Supplementary Table 6.
Validation	All antibodies are commercially available and the validation information found on suppliers' web page is as follows: p-EGFR (suitable for: WB, IHC, IF, F; reacts with: human, mouse, rat, monkey; https://www.cellsignal.com/products/primary-antibodies/phospho-egf-receptor-tyr1068-d7a5-xp-rabbit-mab/3777); EGFR (suitable for: WB, IP, IHC, IF, F; reacts with: human, mouse, monkey; https://www.cellsignal.com/products/primary-antibodies/egf-receptor-d38b1-xp-rabbit-mab/4267); p-RET (suitable for: WB, IP; reacts with: human, D. melanogaster; https://www.cellsignal.com/products/primary-antibodies/phospho-ret-tyr905-antibody/3221); RET (suitable for: WB, IP, IF, F; reacts with: human, mouse; https://www.cellsignal.com/products/primary-antibodies/ret-c31b4-rabbit-mab/3223); p-BRAF (suitable for: WB; reacts with: human, mouse, rat, monkey; https://www.cellsignal.com/products/primary-antibodies/phospho-b-raf-ser445-antibody/2696); BRAF (suitable for: WB, IP, IF, IHC(P, ELISA); reacts with: mouse, rat, human; https://www.scbt.com/p/raf-b-antibody-f-7?productCanUrl=raf-b-antibody-f-7&_requestid=3694081); FGFR3 (suitable for: WB, IP, IF, IHC(P, ELISA); reacts with: human; https://www.scbt.com/p/fgfr-3-antibody-b-9?requestFrom=search); p-ALK (suitable for: WB, IP; reacts with: human; https://www.cellsignal.com/products/primary-antibodies/phospho-alk-tyr1604-antibody/3341); ALK (suitable for: WB, IP, IHC, F; reacts with: human; https://www.cellsignal.com/products/primary-antibodies/alk-d5f3-xp-rabbit-mab/3633); p-MEK (suitable for: WB, IP; reacts with: human, mouse, rat, monkey; https://www.cellsignal.com/products/primary-antibodies/phospho-mek1-2-ser217-221-41g9-rabbit-mab/9154); MEK (suitable for: WB, IP; reacts with: human, mouse, rat, monkey, D. melanogaster; https://www.cellsignal.com/products/primary-antibodies/mek1-2-antibody/9122); p-ERK (suitable for: WB, IP, IHC, IF, FC; reacts with: human, mouse, rat, hamster, monkey, mink, D. melanogaster, zebrafish, bovine, dog, pig, S. cerevisiae; https://www.cellsignal.com/products/primary-antibodies/phospho-p44-42-mapk-erk1-2-thr202-tyr204-d13-14-4e-xp-rabbit-mab/4370); t-ERK (suitable for: WB, IP, IHC, IF, FC; reacts with: human, mouse, rat, hamster, monkey, mink, D. melanogaster, zebrafish, bovine,

dog, pig, S. cerevisiae; https://www.cellsignal.com/products/primary-antibodies/p44-42-mapk-erk1-2-137f5-rabbit-mab/4695); p-AKT (suitable for: WB, IP, IHC, IF, FC; reacts with: human, mouse, rat, hamster, monkey, D. melanogaster, zebrafish, bovine; https://www.cellsignal.com/products/primary-antibodies/phospho-akt-ser473-d9e-xp-rabbit-mab/4060), p-S6 (suitable for: WB, IHC, IF, F; reacts with: human, mouse, rat, monkey, Mink, S. cerevisiae; https://www.cellsignal.com/products/primary-antibodies/phospho-s6-ribosomal-protein-ser235-236-d57-2-2e-xp-rabbit-mab/4858), Cleaved PARP (suitable for: WB, IP, IHC, IF, FC; reacts with: human, monkey; https://www.cellsignal.com/products/primary-antibodies/cleaved-parp-asp214-d64e10-xp-rabbit-mab/5625); BIM (suitable for: WB, IP, IHC, IF, FC; reacts with: human, mouse, rat; https://www.cellsignal.com/products/primary-antibodies/bim-c34c5-rabbit-mab/2933); p-YAP (suitable for: WB, IP, IHC; reacts with: human, mouse, rat; https://www.cellsignal.com/products/primary-antibodies/phospho-yap-ser127-d9w2i-rabbit-mab/13008); YAP (suitable for: WB, IP, IHC, IF, F, ChIP, CUT&RUN; reacts with: human, mouse, rat, Hamster, Monkey; https://www.cellsignal.com/products/primary-antibodies/yap-d8h1x-xp-rabbit-mab/14074); p62 (suitable for: WB, IP, IHC, IF, FC, CoIP, ELISA; reacts with: human, mouse, rat; https://www.ptglab.com/products/SQSTM1-Antibody-18420-1-AP.htm); LC3B (suitable for: WB, IF, F; reacts with: human; https://www.cellsignal.com/products/primary-antibodies/lc3b-d11-xp-rabbit-mab/3868); p-AURKA (suitable for: WB, IF; reacts with: human; https://www.cellsignal.com/products/primary-antibodies/phospho-aurora-a-thr288-c39d8-rabbit-mab/3079); p-MET (suitable for: WB, IP, IHC, IF, F; reacts with: human, mouse, rat; https://www.cellsignal.com/products/primary-antibodies/phospho-met-tyr1234-1235-d26-xp-rabbit-mab/3077); MET (suitable for: WB, IP, IHC, IF, F; reacts with: human; https://www.cellsignal.com/products/primary-antibodies/met-d1c2-xp-rabbit-mab/8198); b-actin (suitable for: WB; reacts with: pig, Hirudo medicinalis, bovine, rat, canine, feline, human, rabbit, carp, mouse, guinea pig, chicken, sheep; https://www.sigmaaldrich.com/US/en/product/sigma/a3854); HSP90 (suitable for: WB, IP, IF, FC, ELISA; reacts with: mouse, rat, human: the vendor cites a paper showing detection of HSP90 by immunoblotting in human neuroblastoma cell lines PMID: # 20655465 Hölzel, M. et al. 2010. Cell. 142: 218-229; https://www.scbt.com/p/hsp-90alpha-beta-antibody-h-114?requestFrom=search)

Eukaryotic cell lines

Policy information about cell lines

Cell line source(s)	The PC-9 cell line was originally established in Tokyo Medical University and obtained from Dr. Nishio Kazuto (Kindai University, Osaka, Japan) in 2005.
Authentication	The PC-9 cell line was confirmed by fingerprinting.
Mycoplasma contamination	All cell lines were periodically tested negative for Mycoplasma throughout the study.
Commonly misidentified lines (See ICLAC register)	None were misidentified.

Human research participants

Policy information about studies involving human research participants

Population characteristics	Using an IRB-approved protocol with written informed consent, a pan-cancer cohort (n=22,742) analyzed by DNA-based hybrid capture NGS OncoPanel at the Dana-Farber Cancer Institute/Brigham and Women's hospital was queried, to find patients with NSCLCs that harbored a sensitizing EGFR mutation as well as fusions. A patient with an EGFR del19 adenocarcinoma which acquired putative GKAP1-NTRK2 fusion detected by hybrid capture RNA-based targeted sequencing in Hospital Sírio-Libanês was included.
Recruitment	Participants at the Dana-Farber Cancer Institute/Brigham and Women's hospital were queried unbiasedly from a pan-cancer cohort. A participant in Hospital Sírio-Libanês was included based on the clinical importance of the detected putative GKAP1-NTRK2 fusion with an activating EGFR del19 mutation.
Ethics oversight	DFCI IRB and the Hospital Sírio-Libanês IRB approved the protocol for this study. Patients provided written informed consent according to CARE guidelines and in compliance with the Declaration of Helsinki principles.

Note that full information on the approval of the study protocol must also be provided in the manuscript.

Flow Cytometry

Plots

Confirm that:

☒ The axis labels state the marker and fluorochrome used (e.g. CD4-FITC).

☒ The axis scales are clearly visible. Include numbers along axes only for bottom left plot of group (a 'group' is an analysis of identical markers).

☒ All plots are contour plots with outliers or pseudocolor plots.

☒ A numerical value for number of cells or percentage (with statistics) is provided.

Methodology

Sample preparation	Cells were washed in PBS+10% FBS, fixed for 10 minutes at room temperature in 2% paraformaldehyde, washed in PBS+10% BS, and permeabilized in cold 90% methanol for 30 minutes on ice; they were then washed and incubated for 1 hour at room

temperature in an anti-RET antibody solution (1:50) in PBS+10% FBS, washed and stained with anti-rabbit Alexa Fluor 488 secondary antibody (1:500; 30 minutes, room temperature). Cells were washed, resuspended in PBS+10% FBS, and analyzed.

Instrument	Samples were analyzed on a BD LSR Fortessa flow cytometer (BD Biosciences).
Software	Data were shown using FlowJo v10 (FlowJo, LLC).
Cell population abundance	Cells (3 x 100,000) were used.
Gating strategy	Gating based on 1) FSC area vs SSC area; 2) FSC height vs width and SSC height vs width were used to gate single cells.

☒ Tick this box to confirm that a figure exemplifying the gating strategy is provided in the Supplementary Information.
